# Role of KLF5 in enhancing ovarian cancer stemness and PARPi resistance: mechanisms and therapeutic targeting

**DOI:** 10.1186/s12967-025-06502-6

**Published:** 2025-04-30

**Authors:** Huimin Xiao, Guiyun Cheng, Haocheng Zhang, Yao Liu, Zhongshao Chen, Yuehan Gao, Feng Gao, Yanling Liu, Shourong Wang, Beihua Kong

**Affiliations:** 1 Department of Obstetrics and Gynecology, Qilu Hospital of Shandong University, Jinan, 250012 China; 2 Gynecologic Oncology Key Laboratory of Shandong Province, Qilu Hospital of Shandong University, Jinan, 250012 China; 3 Qilu Hospital of Shandong University, Jinan, 250012 China

**Keywords:** Ovarian cancer, KLF5, Stemness of cancer cells, PARPi resistance, Vimentin

## Abstract

**Background:**

Ovarian cancer (OC) often presents at advanced stages with poor prognosis. Although poly(ADP-ribose) polymerase inhibitors (PARPi) offer clinical benefits, resistance remains a major challenge. This study investigates the role of KLF5 in regulating OC cell stemness and contributing to PARPi resistance.

**Methods:**

Gene expression analysis was conducted on OC cell lines and their PARPi-resistant counterparts. qRT-PCR and Western blotting assessed the expression levels of stemness markers and KLF5. IHC evaluated KLF5 expression in ovarian cancer tissue samples. Sphere formation and ALDH activity assays were used to evaluate stemness. Chromatin immunoprecipitation (ChIP) investigated KLF5’s binding to the Vimentin promoter. The effects of the KLF5 inhibitor ML264 were tested in vitro using cell viability and apoptosis assays, and in vivo using a xenograft mouse model to evaluate tumor growth and response to PARPi treatment.

**Results:**

PARPi-resistant OC cells showed elevated stemness, indicated by increased SOX2, KLF4, Nanog, and OCT4 expression. KLF5 was significantly upregulated in these cells and linked to poor clinical outcomes. PARPi-resistant cells formed larger and more numerous spheres and had higher ALDH activity. KLF5 bound to the Vimentin promoter, upregulating its expression. Inhibition of KLF5 with ML264 reduced stemness features, decreased Vimentin expression, and resensitized resistant cells to PARPi. In vivo, ML264-treated mice with PARPi-resistant tumors exhibited reduced tumor growth and increased sensitivity to PARPi.

**Conclusion:**

KLF5 enhances stemness and contributes to PARPi resistance in ovarian cancer through Vimentin regulation. Targeting KLF5 offers a promising therapeutic strategy to overcome resistance and improve patient outcomes.

**Supplementary Information:**

The online version contains supplementary material available at 10.1186/s12967-025-06502-6.

## Introduction

Ovarian cancer is the eighth most common cancer in women and is one of the most prevalent malignant tumors of the female reproductive system, accounting for approximately 2.0–2.67% of all new malignant tumor cases in women [1, 2]. It is often challenging to diagnose ovarian cancer at an early stage, resulting in most patients being diagnosed at an advanced stage. This contributes to ovarian cancer being responsible for approximately 3.45–4% of cancer-related deaths among women [1, 2]. High-grade serous ovarian carcinoma (HGSOC) is the most common histological type of ovarian cancer [3], characterized by a high incidence of BRCA mutations (up to about 22%) and homologous recombination repair defects (HRD) (around 50%) [4, 5]. These tumors are more likely to benefit from poly(ADP-ribose) polymerase inhibitors (PARPi), resulting in improvements in progression-free survival (PFS) and overall survival (OS). Olaparib, a first-in-class oral poly(ADP-ribose) polymerase (PARP) inhibitor, functions by trapping PARP1 and PARP2 on DNA at sites of single-strand breaks, thereby preventing DNA repair and leading to synthetic lethality in tumor cells deficient in homologous recombination repair—such as those with BRCA1/2 mutations. As the first PARP inhibitor approved for clinical use, Olaparib has demonstrated significant clinical benefit in patients with high-grade serous ovarian cancer and has become the most widely used PARPi to date. Due to its established efficacy and broad clinical application, Olaparib was selected as the representative PARP inhibitor in this study [6, 7]. However, with the widespread use of PARPi, issues of resistance have become increasingly apparent. Over 40% of patients with BRCA1/2 deficiencies exhibit no response to PARPi [8], and the mechanisms of PARPi resistance are complex and diverse, involving drug target-related mutations (such as PARP1), restoration of homologous recombination, and stabilization of replication forks [9, 10]. 

Cancer stem cells (CSCs) represent a highly valuable research direction in ovarian cancer. These cells possess self-renewal capabilities and tumor-initiating properties, playing a crucial role in tumor proliferation, differentiation, recurrence, metastasis, and chemotherapy resistance [11–13]. Ovarian cancer stem cells are a subgroup of cells within ovarian tumor tissues or the tumor microenvironment, characterized by self-renewal potential, multi-lineage differentiation capabilities, and high tumorigenicity. They often exist in aggregated spheroidal structures and are widely present in malignant ascites associated with ovarian cancer. Increasing evidence suggests that ovarian cancer stem cells can withstand continuous chemotherapy, making recurrent tumors more resistant to treatment and more invasive [14, 15], serving as major drivers of malignant tumor growth, drug resistance, metastasis, and recurrence [16–18]. KLF5 (Kruppel-like factor 5) is a member of the Kruppel-like factor family and acts as a pluripotency factor that regulates cell lineage and stemness [19, 20]. It is expressed in stem cells and participates in various cellular functions, with downstream targets such as Nanog, SOX2, and OCT4 playing key roles in maintaining cellular pluripotency [21]. Studies have indicated that KLF5 is abnormally expressed in various solid tumors, including breast cancer, prostate cancer, colon cancer, non-small cell lung cancer, and esophageal squamous cell carcinoma, driving the progression and metastasis of these tumors [22]. As a selective inhibitor of KLF5, ML264 exhibits potent antitumor activity in multiple preclinical models including colorectal and ovarian cancers, and possesses favorable pharmacokinetic properties with promising safety for KLF5-targeted therapeutic strategies [23, 24]. 

Recent studies have demonstrated that KLF5 forms a transcriptional complex with EHF and ELF3, remodeling RAD51 transcription to enhance the homologous recombination repair (HRR) pathway in ovarian cancer cells, thereby promoting ovarian cancer progression and PARPi resistance [25]. In this study, we confirmed the regulatory role of KLF5 in ovarian cancer cell stemness, focusing on the mechanisms by which this process contributes to PARPi resistance in ovarian cancer. We also conducted preclinical explorations targeting KLF5 to reverse PARPi resistance.

## Methods

### Cell lines and cell culture

The SKOV3 cells and HEK293T cells were purchased from the Chinese Academy of Sciences (Shanghai, China). OV90,COV362 and ES2 cell lines were obtained from the American Type Culture Collection. A2780 and OVCAR8 cell lines came from the M.D Anderson Cancer Center characterized Cell line Core. Hey cells were derived from Dr.Liu’s laboratory. HOC7 cell line was a gift from Dr.R.N.Buick (University of Arizona, Tucson). PEO14 was a gift from Dr.S.P.Langdon (ICRF, UK). Olaparib-resistant A2780 (A2780-olaR) cell line were obtained from Dr.Ma’s laboratory (Huazhong University of Science and Technology). SKOV3-olaR cells were induced by exposing SKOV3 cells to gradually increasing concentrations of olaparib in our lab. All cell lines were validated by short tandem repeat (STR) profiling and mycoplasma testing. SKOV3, Hey, COV362, ES2 cell lines were cultured in Dulbecco’s modified Eagle’s medium (DMEM) supplemented with 10% FBS. A2780, HOC7, PEO14 cells were cultured in PRIM1640 medium containing 10% FBS. OV90 and HEK293T were cultured in DMEM plus 15% FBS. All cells were sustained in a humidified incubator with 95% O_2_ and 5% CO_2_ at 37℃.

### Patients and tumor samples

Ovarian cancer tissue and fallopian tube tissue were obtained from the pathological tissue specimen bank of Obstetrics and Gynecology, Qilu Hospital, Shandong University.The ethics application has been approved by the Ethics Committee of Qilu Hospital.

### RNA interference and lentiviral infection

Small interfering RNA sequences targeting KLF5 and Vimentin were purchased from GenePharma (Shanghai, China). The sequential transfection of siRNA was performed according to the Lipofectamine 2000(Invitrogen, USA) instructions. The sequence of KLF5 overexpression and knockdown were cloned into pLVX-Puro and pLKO.1-EGFP-Puro vectors, respectively. Vimentin overexpression plasmid from TSINGKE was cloned into pcDNA3.1 vector. The plasmid to be transfected, psPAX2 and pMD2.G, were transfected into HEK293T cells in Lipofectamine 2000 to obtain virus particles. Ovarian cancer was infected by the collected viral fluid. The stable transfected cell lines were selected with puromycin(Merck Millipore, USA) for two weeks.

### Drug preparation and usage

Olaparib (Catalog No. S1060), ML264 (Catalog No. S8196) and Veliparib (Catalog No. S1004) were purchased from Selleck Chemicals, and EB-47 (Catalog No. HY-108631) was obtained from MedChemExpress. Stock solutions of Olaparib, ML264 and Veliparib were prepared in 100% DMSO at a concentration of 50 mM, while EB-47 was dissolved in 100% DMSO at 10 mM. All stock solutions were stored at − 20 °C and diluted in culture medium to the desired working concentrations before use. For cell viability assays, Olaparib, Veliparib, and EB-47 were used at concentrations ranging from 0 to 100 µM. Olaparib was used in additional colony formation and in vivo xenograft experiments. In colony formation assays, the final concentration of Olaparib was 200 nM. For in vivo experiments, Olaparib was administered at 50 mg/kg via intraperitoneal injection every two days, prepared in a vehicle consisting of 10% DMSO, 40% PEG300, 5% Tween-80, and 45% ddH₂O.

### Colony formation assay

A2780, A2780-olaR, SKOV3 and SKOV3-olaR (500 cells per well) were planted into a six-well plate treated with the corresponding concentration of olaparib. After treatment for 10–14 days, the cells were immobilized with methanol and stained with 0.1% crystal violet. The number of clones was counted and three independent experiments were performed.

### Cell viability analysis

Ovarian cancer cells were seeded in 96-well plates (3000–5000 cells per well) and treated with gradient concentrations of olaparib or ML264. Fresh medium containing 10% CCK-8 solution (Vazyme, China) was added to each well, and the same solution was added to blank wells as a control and incubated at 37℃ for 3 h. The absorbance of the sample was measured at 450 nm by a microplate spectrophotometer (BioRad, USA).

### Sphere-formation assay

The cells were digested, centrifuged and collected, and washed twice with PBS. Cells were resuspended in freshly equipped stem cell medium. 10ul pre-cooled cell suspension was mixed with 10ul Matrigel glue 1:1 and spread on 12-well ultra-low adhesion plate. After 30 min of solidification in 37℃ incubator, each well was cultured with 200ul stem cell medium. The number of spheres formed was counted and photographed after 2 weeks.

### Flow cytometry

Cell apoptosis was detected by flow cytometry. The cells were digested and washed with PBS and then suspended in 1x Binding Buffer (BD BioScience, USA). The samples were stained for 10 min by 5ul FITC-Annexin V and 5ul Propidium iodide(PI). The proportion of apoptotic cells was detected by BD FACSCalibur Flow cytometer. Data analysis was performed with Flow Jo V10.4. ALDEFLUOR assay was performed according to the instructions of the ALDEFLUORTM Kit (Stemcell, Canada).

### Western blot

Cell precipitate was collected, lysed by RIPA buffer with PMSF (Beyotime, China) and ultrasonically lysed on ice. The protein concentration was measured by a BCA protein assay kit (Merck Millipore, USA). Protein samples were isolated by SDS-PAGE electrophoresis and then transferred to PVDF membranes (Merck Millipore, USA). Nonspecific antigens on PVDF were blocked with skim milk at room temperature and incubated with primary antibodies at 4 ℃ overnight. Next day, the PVDF membrane was washed by TBST every 5 min for a total of 6 times. The membrane was incubated with HRP-conjugated secondary antibodies for 1.5 h and washed with TBST again for 6 times. An ECL system (GE Health Care) was used to detect the protein expression of the samples, and GAPDH/β-actin was used as an endogenous control. All antibodies information is shown in supplemental Table 1.

### Immunohistochemical staining

High-grade serous ovarian cancer tissue microarrays were obtained from the Tissue Specimen Bank of the Department of Obstetrics and Gynecology, Qilu Hospital, Shandong University. Immunohistochemical staining was performed on the tissue microarray and mouse tumor tissues using a General Two-step Detection Kit (Zhongshan Biotechnology Company, China) according to the manufacturer’s protocol. Tissue slides were deparaffinized, hydrated, antigen repaired with EDTA, endogenous peroxidase blocked, and incubated with primary antibodies overnight at 4 ℃. Primary antibodies include KLF5 (1:50), Vimentin (1:50), KLF4 (1:50), and Ki67 (5ug/ml). The antigen-antibody response signal was enhanced by the reaction enhancer solution and incubated with enzyme-labeled sheep anti-mouse/rabbit IgG polymer. The DAB Test Kit (Zhongshan Biotechnology Company, China) were stained to detect gene expression. Slides were observed microscopically and photographed.The slides were blindly scored by two expert pathologists based on the intensity and extent of staining. The staining intensity was divided into 0 (negative), 1 (weak), 2 (moderate) and 3 (strong). The staining extent was 0 (0), 1 (1–25%), 2 (26–50%), 3 (51–75%), 4 (76–100%).The final score was obtained by multiplying the staining intensity by the staining extent. Each sample had two replicates, and the final score was the average score of the replicates. Each sample was scored as low expression if the final score was less than or equal to 7 and high expression if the final score was greater than 7. Ki67 was scored according to the positive rate.

### qPCR

Total RNA was extracted from samples using TRIzol reagent(Invitrogen, USA). RNA concentration was measured using a NanoVue Plus ultramicrospectrophotometer (GE, USA). The RNA was reverse-transcribed into cDNA by the HiScript III RT SuperMix. qPCR was performed with the SYBR Green Premix Ex Taq (Takara, Japan) kit. The relative RNA expression levels of samples were detected by the Applied Biosystems QuantStudio3 and calculated by the 2^−ΔΔCT^ method. The primers of qPCR were shown in Supplementary Table 2.

### Luciferase assay

KLF5 overexpression A2780 cell line and the control were implanted in a 12-well plate. The plasmid containing the promoter sequence and the pRL-TK plasmid were co-transfected. The absorbance of firefly luciferase and renila luciferiase was detected with the Dual Luciferase Reporter Assay kit (Vazym, China) by a microplate spectrophotometer (BioRad, USA).

### RNA-seq analysis

KLF5 knowdown SKOV3 cells and control cells were collected and performed the next-generation sequencing by the Novogene Bio-informatics Company. The RNA-seq data has been submitted to NCBI GEO database and the accession number was GSE248659. The expression threshold of differential genes was set as log2 Fold change >1 or <-1 and *p* < 0.05. GO analysis was performed with R package ClusterProfiler. Enrichr analysis was performed with Erichr website (https://maayanlab.cloud/Enrichr/).

### Chromatin immunoprecipition(ChIP) and ChIP-qPCR

The sample was fixed with 1% formaldehyde at room temperature, and the protein and DNA were reversibly cross-linked. The cross-linking reaction was terminated by 125mM glycine, and the samples were lysed by the nuclear lysate at room temperature and then ultrasonically broken by a Bioruptor UCD-200 (Diagenode, Liege, Belgium). The ultrasonic lysate was immunoprecipitated by Protein G beads and KLF5 antibody or corresponding control IgG. Protein G beads after IP was washed successively with different ionic strength buffers. The protein-DNA complex was eluted from the beads for decross-linking and DNA purification. The primers of ChIP-qPCR were shown in Supplementary Table 2.

### Mouse tumor model

All procedures of animal experiments have been approved by the Animal Experiment Ethics Committee of Qilu Hospital of Shandong University. 1 × 10^6^ ovarian cancer cells were subcutaneously injected into the flank of BALB/c nude mice. 24 nude mice were randomly divided into 4 groups (6 mice per group). When the tumor volume reached 50–100 mm^3^, the corresponding treatments were given: Vehicle (10%DMSO), Olaparib (50 mg/kg), ML264 (10 mg/kg), combined usage of Olaparib and ML264. The drug was administered intraperitoneally every 2 days and the volume of the tumor were measured.Then mice were euthanized and tumors were dissected, photographed, weighed, and measured.

### Tunel assay

The tumor tissues of mice were prepared into paraffin sections, and then apoptosis was detected according to the instructions of the TMR (Red) Tunel Cell Apotosis Detection kit (Servicebio, China). The tissue slides were dewaxed, permeated with protease K and washed with PBS for three times. After equilibration with Equilibration Buffer, the slides were incubated with TdT incubation buffer at 37 ℃ for 1 h and washed three times with PBS again. Slides were observed under a fluorescence microscope immediately after staining with DAPI solution and sealing with anti-fluorescence quench.

### Drug combination analysis

A2780-olaR and SKOV3-olaR cells were treated with olaparib and ML264 in combination at different drug concentrations. 72 h after administration, OD values at 450nm were measured by CCK8 assay. Drug combination analysis was developed by CompuSyn software 1.2.0. Combinatorial effects are defined by combination index (CI) values with synergistic (< 1), additive (= 1), and antagonistic (> 1) effects.

### Statistical analysis

All data in this study was conducted by Graphad Prism 9. The Log-rank test was used to analyze differences in clinical outcomes. The Student’s t-test was used to determine the significance of the difference between two groups. Significant differences in variables were analyzed in One-way ANOVA between multiple groups. The mean and standard deviation were determined by three independent repetitions. *P* < 0.05 was taken as the threshold of statistical significance (* *P*<0.05, **P<0.01, ***P<0.001).

## Results

### PARPi-resistant ovarian cancer cells exhibit enhanced stemness

**Fig. 1:**
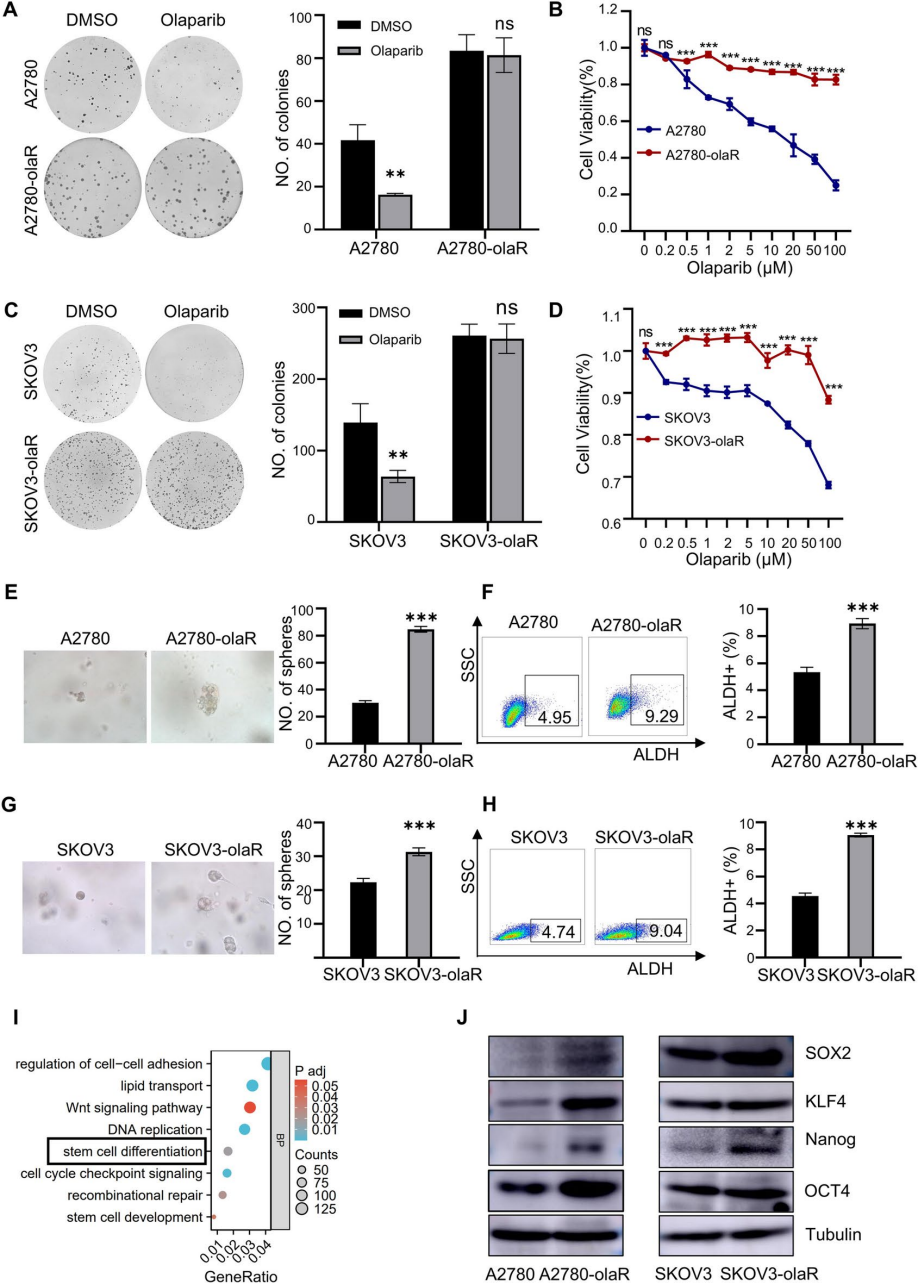
PARPi-resistant ovarian cancer cells obtain increased stemness. **A** Clonogenic assay of A2780 and A2780-olaR cells with DMSO or olaparib. **B** Cell viability curve of A2780 and A2780-olaR cells exposed to different concentration of olaparib for 72 h. **C** Clonogenic assay of SKOV3 and SKOV3-olaR cells with DMSO or olaparib. **D** Cell viability curve of SKOV3 and SKOV3-olaR cells exposed to different concentration of olaparib for 72 h. **E** Representative pictures and statistical results of A2780 and A2780-olaR cell spheroidization. **F** Flow chart and statistical diagram of ALDH-positive cells in A2780 and A2780-olaR cells. **G** Representative pictures and statistical results of SKOV3 and SKOV3-olaR cell spheroidization. **H** Flow chart and statistical diagram of ALDH-positive cells in SKOV3 and SKOV3-olaR cells. **I** GO analysis of RNA expression of A2780-olaR compared to A2780 based on GSE153867 data set. **J** Western blot image of SOX2, KLF4, Nanog and OCT4 protein expression in A2780, A2780-olaR, SKOV3 and SKOV3-olaR cells. *P* value was obtained by Student’s t-test. Results represent the mean ± SD. *P < 0.05, **P < 0.01, ***P < 0.001

The plate cloning assay demonstrated a significant growth inhibition in ovarian cancer cell lines A2780 and SKOV3 following treatment with olarparib, whereas PARPi-resistant cells A2780-olaR and SKOV3-olaR exhibited no significant difference compared to the control group (Fig. 1A and C). Additionally, the CCK8 assay revealed a notable decrease in cell viability of A2780 and SKOV3 cell lines at lower concentrations, while the cell viability of A2780-olaR and SKOV3-olaR cell lines remained largely unaffected at the same concentration (Fig. 1B and D). Subsequently, we analysed the high-throughput sequencing data conducted on A2780-olaR and its control cell A2780 (GSE153867) to investigate the mechanism of PARPi resistance. The RNA-seq results revealed a significant enrichment of stem cell differentiation pathways in A2780-olaR cells (Fig. 1I). Additionally, the sphere-forming ability of A2780-olaR, as determined by 3D culture technology, was notably higher than that of A2780 (Fig. 1E), and similarly, the sphere-forming ability of SKOV3-olaR was significantly greater than that of SKOV3 (Fig. 1G). It is important to note that ALDH serves as a universal marker of tumor cell stemness. The percentage of ALDH-positive cells in ovarian cancer cell lines as determined by flow cytometry exhibited a significant increase in A2780-olaR (Fig. 1F) and SKOV3-olaR (Fig. 1H) cell lines compared to that in A2780 and SKOV3 cell lines, suggesting an augmentation in the stemness of PARPi-resistant cells. Additionally, western blot analysis revealed elevated levels of SOX2, KLF4, Nanog, and OCT4 in the PARPi-resistant cells A2780-olaR and SKOV3-olaR (Fig. 1J).

### KLF5 is associated with PARPi resistance and unfavorable prognosis in ovarian cancer

**Fig. 2:**
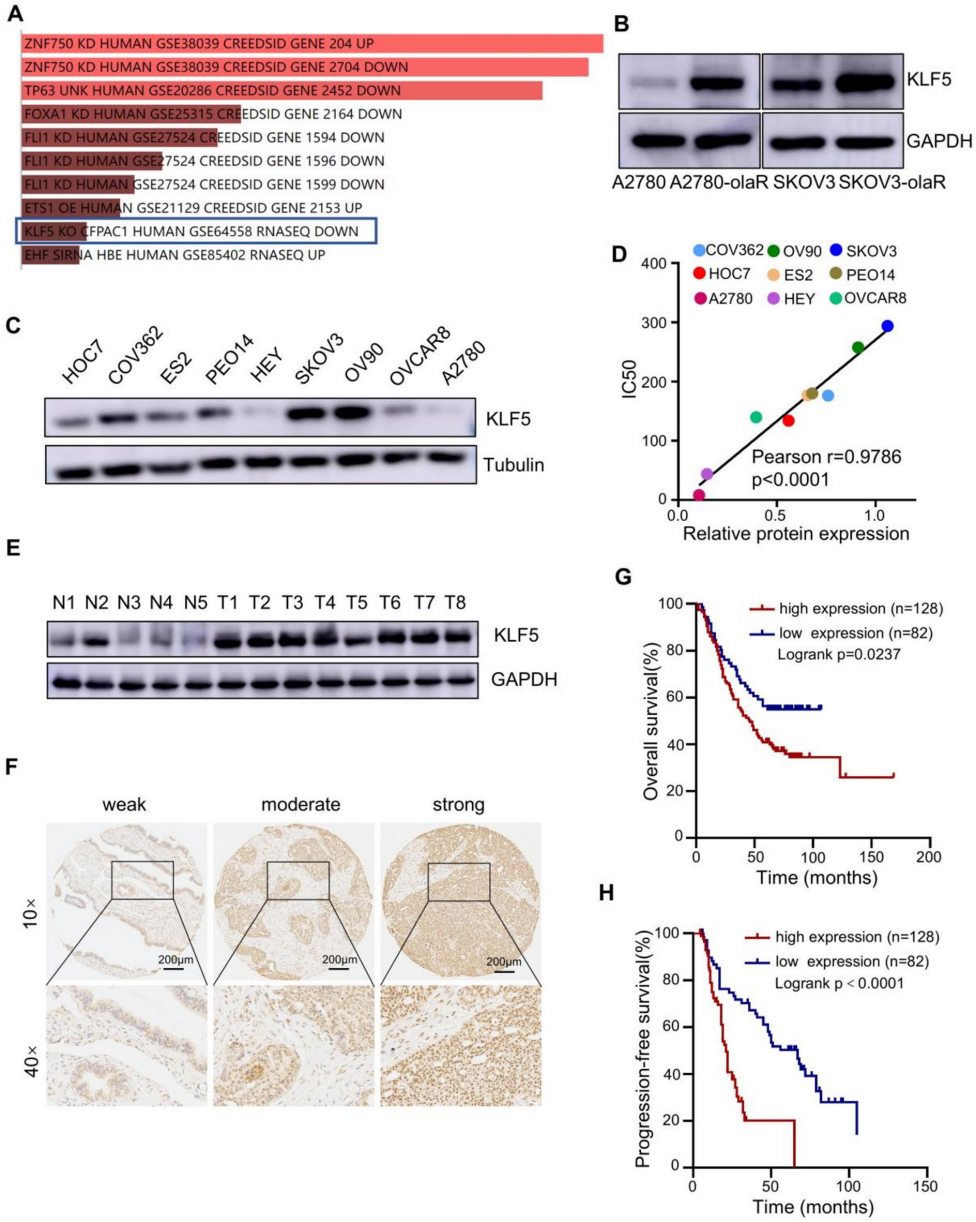
KLF5 is related to PARPi resistance and poor prognosis of ovarian cancer. **A** Enrichr analysis of genes highly expressed in A2780-olaR cells compared to A2780 cells based on GSE153867 data set. **B** Western blot image of KLF5 protein expression in A2780, A2780-olaR, SKOV3 and SKOV3-olaR cells. **C** KLF5 protein expression in different ovarian cancer cells. **D** Correlation analysis between KLF5 protein expression and olaparib IC50 in different ovarian cancer cells. **E** KLF5 protein expression detected by western blot in ovarian cancer and fallopian tube tissues. N1-N5 represent normal fallopian tube tissues obtained from patients who underwent hysterectomy and salpingectomy for benign uterine diseases. T1-T8 represent ovarian cancer tumor tissues. **F** Representative immunohistochemistry images of KLF5 expression in ovarian cancer tissue microarrays from Qilu Hospital. **G** Overall survival analysis of ovarian cancer tissue microarrays from Qilu Hospital, grouped according to KLF5 expression. **H** Progression-free survival analysis of ovarian cancer tissue microarrays from Qilu Hospital, grouped according to KLF5 expression. *P* value was obtained by Log-rank test

Differential gene expression analysis was conducted on A2780 and A2780-olaR cells based on GSE153867 data set to identify key genes in PARPi resistance. The Enrichr analysis revealed that KLF5 functions as a crucial transcription factor in promoting PARPi resistance in ovarian cancer, as depicted in Fig. 2A. Additionally, the protein expression of KLF5 was found to be elevated in PARPi-resistant cell lines A2780-olaR and SKOV3-olaR compared to their sensitive counterparts A2780 and SKOV3, as illustrated in Fig. 2B. The western blot technique was utilized to assess the expression levels of KLF5 in nine ovarian cancer cell lines, revealing that the SKOV3 cell line exhibited the highest expression while the A2780 cell line displayed the lowest (Fig. 2C). The IC50 values of these cell lines were determined through CCK8 assay, and a positive correlation between IC50 and KLF5 expression levels was observed, with a Pearson correlation coefficient of 0.9786 and a significance level of *P* < 0.0001 (Fig. 2D). Subsequently, in an effort to investigate the expression of KLF5 in ovarian cancer and its correlation with patient prognosis, western blot and tissue immune microarray analysis was conducted on ovarian cancer tissues obtained from the Obstetrics and Gynecology Department of Qilu Hospital at Shandong University using immunohistochemical staining techniques (Fig. 2E and F). As anticipated, Western blot results demonstrated that KLF5 expression was higher in ovarian cancer tissues compared to normal fallopian tube tissues (Fig. 2E) and high KLF5 expression levels demonstrated correlations with both poorer overall survival (Fig. 2G) and progression-free survival (Fig. 2H) in ovarian cancer patients. These findings indicate a potential link between elevated KLF5 expression and unfavorable outcomes in ovarian cancer patients.

### Inhibition of KLF5 decreases the stemness of ovarian cancer cells

**Fig. 3:**
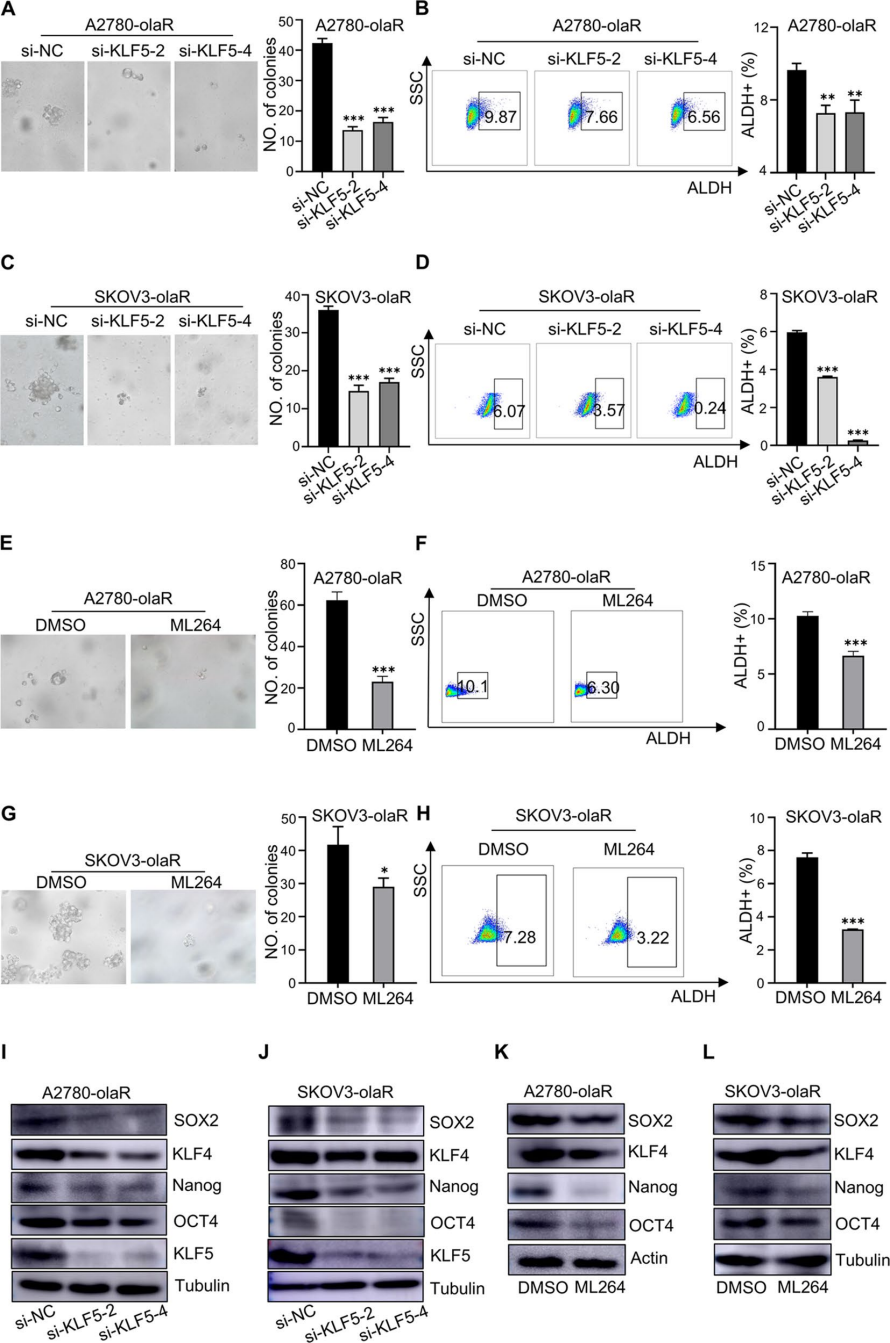
Inhibiting KLF5 reduces the stemness of ovarian cancer. **A** Representative pictures and statistical diagram of different KLF5 knock down A2780-olaR cells and corresponding control cell spheroidization. **B** Flow chart and statistical diagram of ALDH-positive cells in different KLF5 knock down A2780-olaR cells and corresponding control cells. **C** Representative pictures and statistical diagram of different KLF5 knock down SKOV3-olaR cells and corresponding control cell spheroidization. **D** Flow chart and statistical diagram of ALDH-positive cells in different KLF5 knock down SKOV3-olaR cells and corresponding control cells. **E** Representative pictures and statistical diagram of A2780-olaR cell spheroidization with DMSO or ML264. **F** Flow chart and statistical diagram of ALDH-positive cells in A2780-olaR cells with DMSO or ML264. **G** Representative pictures and statistical diagram of SKOV3-olaR cell spheroidization with DMSO or ML264. **H** Flow chart and statistical diagram of ALDH-positive cells in SKOV3-olaR cells with DMSO or ML264. **I** Western blot image of SOX2, KLF4, Nanog, OCT4 and KLF5 protein expression in different KLF5 knock down A2780-olaR cells and corresponding control cells. **J** Western blot image of SOX2, KLF4, Nanog, OCT4 and KLF5 protein expression in different KLF5 knock down SKOV3-olaR cells and corresponding control cells. **K** Western blot image of SOX2, KLF4, Nanog, OCT4 and KLF5 protein expression in A2780-olaR cells with DMSO or ML264. **L** Western blot image of SOX2, KLF4, Nanog, OCT4 and KLF5 protein expression in SKOV3-olaR cells with DMSO or ML264. *P* value was obtained by Student’s t-test and ANOVA analysis. Results represent the mean ± SD of three independent experiments. *P < 0.05, **P < 0.01, ***P < 0.001

To investigate the function of KLF5 further, we utilized the pLVX vector to establish KLF5-overexpressing cell lines of OVCAR8 and A2780, along with their respective control cells, and confirmed the efficacy of the overexpression.The expression of KLF5 at the protein and mRNA levels were significantly elevated in OVCAR8-KLF5 (SupFig. 1A and SupFig. 1B) and A2780-KLF5 (SupFig. 1C and SupFig. 1D). To suppress KLF5 expression, four distinct small interference RNA sequences were transfected in A2780-olaR (SupFig. 1E and SupFig. 1F) and SKOV3-olaR (SupFig. 1G and SupFig. 1H), with subsequent validation of protein and mRNA levels. Among the sequences tested, si-KLF5-2 and si-KLF5-4 demonstrated the most pronounced knockdown efficiency, prompting their selection for further functional experiments. Following KLF5 silencing using small interference RNA, the sphere forming assay revealed a decrease in both the volume and quantity of ovarian cancer cells (Fig. 3A and C). Additionally, the percentage of ALDH positive cells in ovarian cancer cells, as measured by flow cytometry, was significantly reduced after KLF5 knock down (Fig. 3B and D). Conversely, KLF5-overexpressing cells exhibited an increase in the volume and number of spheres formed (SupFig. 2A and SupFig. 2D).Flow cytometry results indicated a significant increase in the proportion of ALDH positive cells in ovarian cancer cell lines that overexpressed KLF5 (SupFig. 2B and SupFig. 2E). Subsequently, the markers of stemness measured by western blot showed a decrease following KLF5 knock down and an increase following KLF5 overexpression (Fig. 3I, J and SupFig. 2C, F). Moreover, administration of A2780-olaR and SKOV3-olaR with ML264 for KLF5 inhibition resulted in a reduction in the sphere-forming ability of ovarian cancer cells (Fig. 3E and G).Flow cytometry analysis revealed a significant reduction in the proportion of ALDH positive cells in ovarian cancer cells treated with ML264 (Fig. 3F and H). And the markers of stemness was reduced after ML264 treatment (Fig. 3K, L). These results demonstrate that KLF5 is associated with the stemness of ovarian cancer.

### Inhibition of KLF5 enhances PARPi sensitivity in PARPi-resistant ovarian cancer cells but has minimal effect in PARPi-sensitive cells

**Fig. 4:**
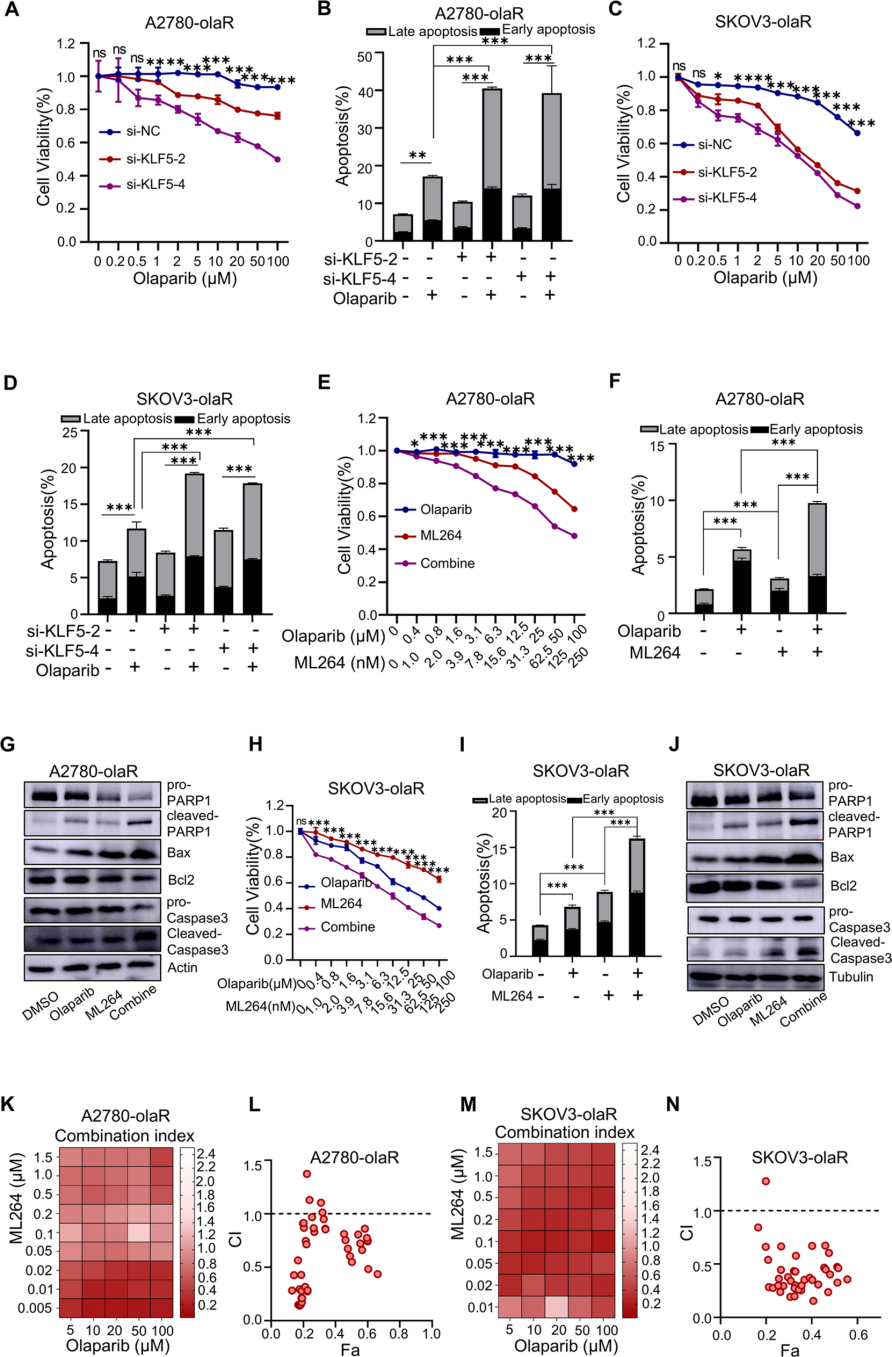
Inhibiting KLF5 improves PARPi sensitivity in PARPi-resistant ovarian cancer. **A** Cell viability curve of different KLF5 knock down A2780-olaR cells and corresponding control cells exposed to different concentrations of olaparib for 72 h. **B** Statistical diagram of apotosis cells in different KLF5 knock down A2780-olaR cells and corresponding control cells with DMSO or olaparib. **C** Cell viability curve of different KLF5 knock down SKOV3-olaR cells and corresponding control cells exposed to different concentrations of olaparib for 72 h. **D** Statistical diagram of apotosis cells in different KLF5 knock down SKOV3-olaR cells and corresponding control cells with DMSO or olaparib. **E** Cell viability curve of A2780-olaR cells exposed to different concentrations of olaparib, ML264 and the combination for 72 h. **F** Statistical diagram of apotosis cells in A2780-olaR cells exposed to olaparib, ML264 and the combination for 72 h. **G** Western blot image of pro-PARP1 (the full-length PARP1 used for background quantification of Cleaved PARP1) and cleaved-PARP1, Bax, Bcl2, pro-caspase3 and cleaved-caspase3 protein expression in A2780-olaR cells exposed to olaparib, ML264 and the combination. **H** Cell viability curve of SKOV3-olaR cells exposed to different concentration of olaparib, ML264 and the combination for 72 h. **I** Statistical diagram of apotosis cells in SKOV3-olaR cells exposed to olaparib, ML264 and the combination for 72 h. **J** Western blot image of pro-PARP1 and cleaved-PARP1, Bax, Bcl2, pro-caspase3 and cleaved-caspase3 protein expression in SKOV3-olaR cells exposed to olaparib, ML264 and the combination. **K** Heatmap of combination index (CI) values for combination treatment between olaparib and ML264 in A2780-olaR. **L** CI values for the entire fraction affected (Fa) of A2780-olaR. **M** Heatmap of combination index (CI) values for combination treatment between olaparib and ML264 in SKOV3-olaR. **N** CI values for the entire fraction affected (Fa) of SKOV3-olaR. *P* value was obtained by ANOVA analysis. Results represent the mean ± SD of three independent experiments. *P < 0.05, **P < 0.01, ***P < 0.001

Following KLF5 knockdown using si-RNA, CCK8 analysis revealed a significant reduction in the cell viability of A2780-olaR and SKOV3-olaR exposed to different concentrations of olaparib (Fig. 4A and C). A similar trend was also observed when cells were treated with other PARP inhibitors, including Veliparib and EB-47 (SupFig. 5). Conversely, overexpression of KLF5 led to a marked increase in the cell activity of PARPi-sensitive A2780 and OVCAR8 cells exposed to different concentrations of olaparib (SupFig. 3A and SupFig. 3C). Flow cytometry analysis was utilized to assess the apoptosis ratio of A2780-olaR and SKOV3-olaR cells following treatment with DMSO or olaparib for 72 h.The findings indicated an increase in the apoptosis ratio of KLF5-knock down A2780-olaR and SKOV3-olaR cells following treatment with olaparib (Fig. 4B and D). Additionally, flow cytometry analysis revealed a reduction in the proportion of apoptosis in the KLF5-overexpressed A2780 and OVCAR8 cell lines following olaparib treatment compared to the control group (SupFig. 3B and SupFig. 3D). The findings suggest that downregulation of KLF5 can enhance the susceptibility of PARPi-resistant ovarian cancer cells to PARP inhibitors, while upregulation of KLF5 can promote resistance of PARPi-sensitive ovarian cancer cells to PARP inhibitors. We then evaluated whether pharmacological inhibition of KLF5 would restore the antitumor effect of olaparib on PARPi-resistant ovarian cancer cells. To this end, we combined olaparib with ML264 [24], a specific KLF5 inhibitor previously described in multiple cancer models. we treated the cells with a fixed ratio of olaparib and ML264 (1:2.5) at a series of concentrations. In A2780-olaR and SKOV3-olaR cells, coadministration of olaparib and ML264 showed more potent inhibition of cell viability than either drug alone (Fig. 4E and H). Flow cytometry confirmed that ML264 significantly enhanced olaparib-induced apoptosis in A2780-olaR and SKOV3-olaR cells (Fig. 4F and I). Subsequent western blot analysis showed the highest expression of pro-apoptotic Bax, cleaved PARP1 and cleaved-caspase3 and lowest expression of anti-apoptotic Bcl2 upon coadministration of olaparib and ML264 in A2780-olaR and SKOV3-olaR cells (Fig. 4G and J). To further explore the synergistic effect of olaparib and ML264, we designed a matrix of concentrations, wherein multiple combinatorial ratios reached significant synergy in both A2780-olaR and SKOV3-olaR cells characterized by the combination index (CI) < 1 (Fig. 4K, L,M, N). Together, these results demonstrate that olaparib and ML264 combination leads to synergistic inhibition on the viability of PARPi-resistant ovarian cancer cells.

To further investigate whether the sensitization effect of KLF5 inhibition on PARPi is specifically observed in PARPi-resistant cells, we performed genetic and pharmacological inhibition of KLF5 in PARPi-sensitive A2780 and OVCAR8 cells. The knockdown efficiency of KLF5 by siRNA is shown in SupFig. 4A. CCK8 results indicate that in OVCAR8 cells treated with a series of concentrations of olaparib, KLF5 knockdown led to a slight reduction in cell viability (SupFig. 4B). In A2780 cells, however, KLF5 knockdown had no effect on cell viability (SupFig. 4F). In line with this, in A2780 and OVCAR8 cells, pharmacological inhibition of KLF5 by ML264 combined with olaparib had similar effects on cell activity as olaparib alone (SupFig. 4C and SupFig. 4G). Moreover, the results of the concentration matrix showed that the combination index (CI) of olaparib and ML264 is near 1 in A2780 and OVCAR8 cells (SupFig. 4D, E, H, I). In summary, the above results suggest that inhibition of KLF5 enhances PARPi sensitivity in PARPi-resistant ovarian cancer cells but has minimal effect in PARPi-sensitive cells.

### ML264 enhances the sensitivity of PARPi-resistant ovarian cancer cells to olaparib in vivo

**Fig. 5:**
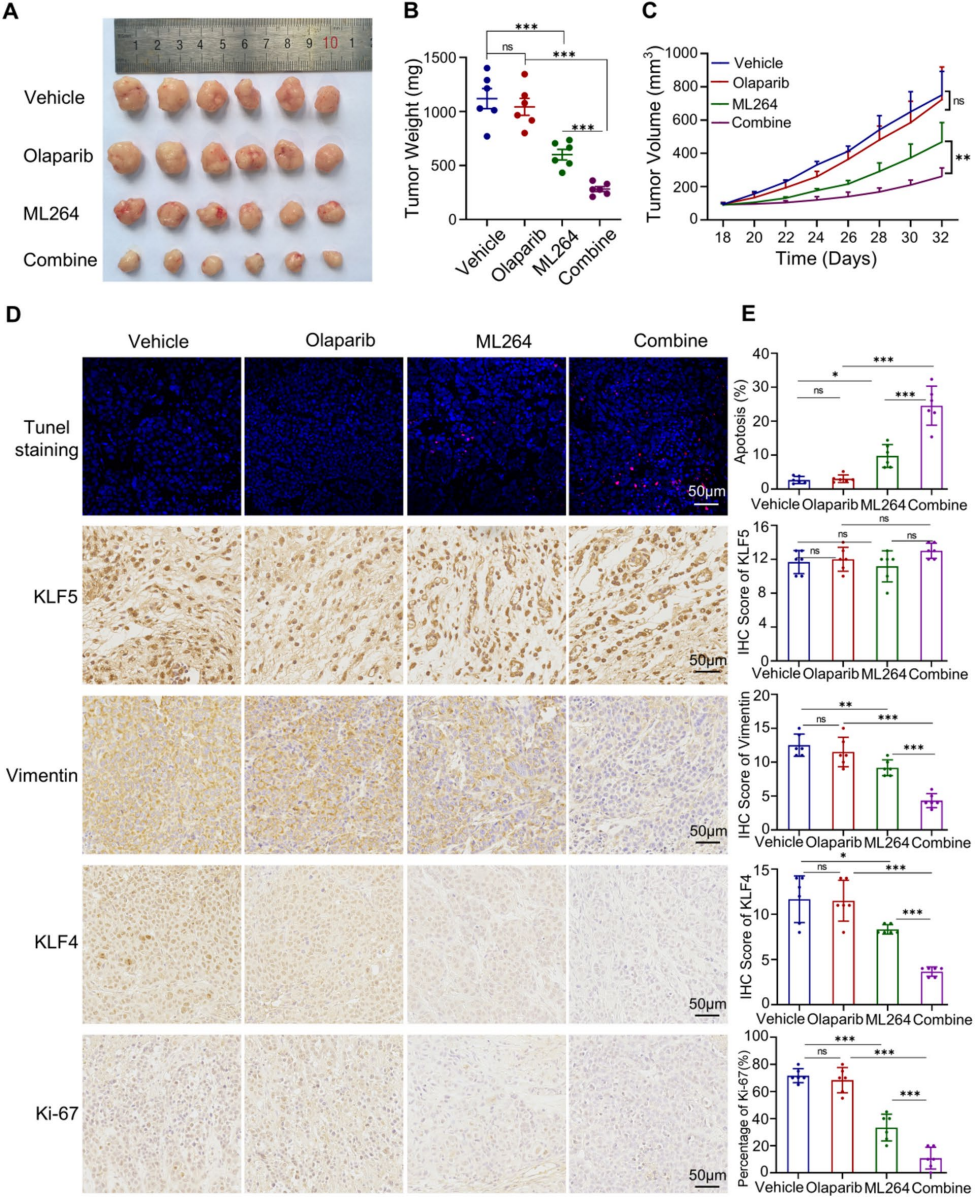
ML264 enhances the sensitivity of PARPi-resistant ovarian cancer cells to olaparib in vivo. **A**-**C**. Image of xenograft tumors, tumor mass, and tumor volume in mice subcutaneously injected with A2780-olaR cells treated with DMSO, olaparib, ML264, or combination. **D**. Image of Tunel staining and immunohistochemical staining of KLF5, Vimentin, KLF4 and Ki-67 in xenograft tumor in nude mice subcutaneously injected with A2780-olaR cells treated with DMSO, olaparib, ML264, or combination. **E**. Statistical diagram of proportion of apoptosis and protein expression of KLF5, Vimentin, KLF4 and Ki-67 in xenograft tumor in nude mice subcutaneously injected with A2780-olaR cells treated with DMSO, olaparib, ML264, or combination. P value was obtained by ANOVA analysis. Results represent the mean ± SD of six independent experiments. *P < 0.05, **P < 0.01, ***P < 0.001

In order to investigate the antitumor effects of ML264 on PARPi-resistant ovarian cancer cells in vivo, A2780-olaR cells were subcutaneously inoculated into immunodeficient BALB/c-nude mice, which were then treated with DMSO, Olaparib, ML264, or a combination of both. Interestingly, ML264 alone significantly inhibited tumor growth, whereas olaparib alone did not have a significant impact, and the combination of ML264 and olaparib demonstrated a more potent inhibitory effect than either one (Fig. 5A-C). Consistently, tunel staining revealed a most significant increase in apoptotic cell ratio following treatment with combination of olaparib and ML264 than either one (Fig. 5D, E). Immunohistochemical analysis demonstrated no significant difference in KLF5 expression among the four groups (Fig. 5D, E). Conversely, the expression of Vimentin and KLF4 and the positive proportion of Ki-67 positive cells decreased most significantly in the combination group (Fig. 5D, E). These findings suggest that ML264 enhances the sensitivity of PARPi-resistant ovarian cancer to olaparib in vivo.

### KLF5 regulates the expression of Vimentin

**Fig. 6:**
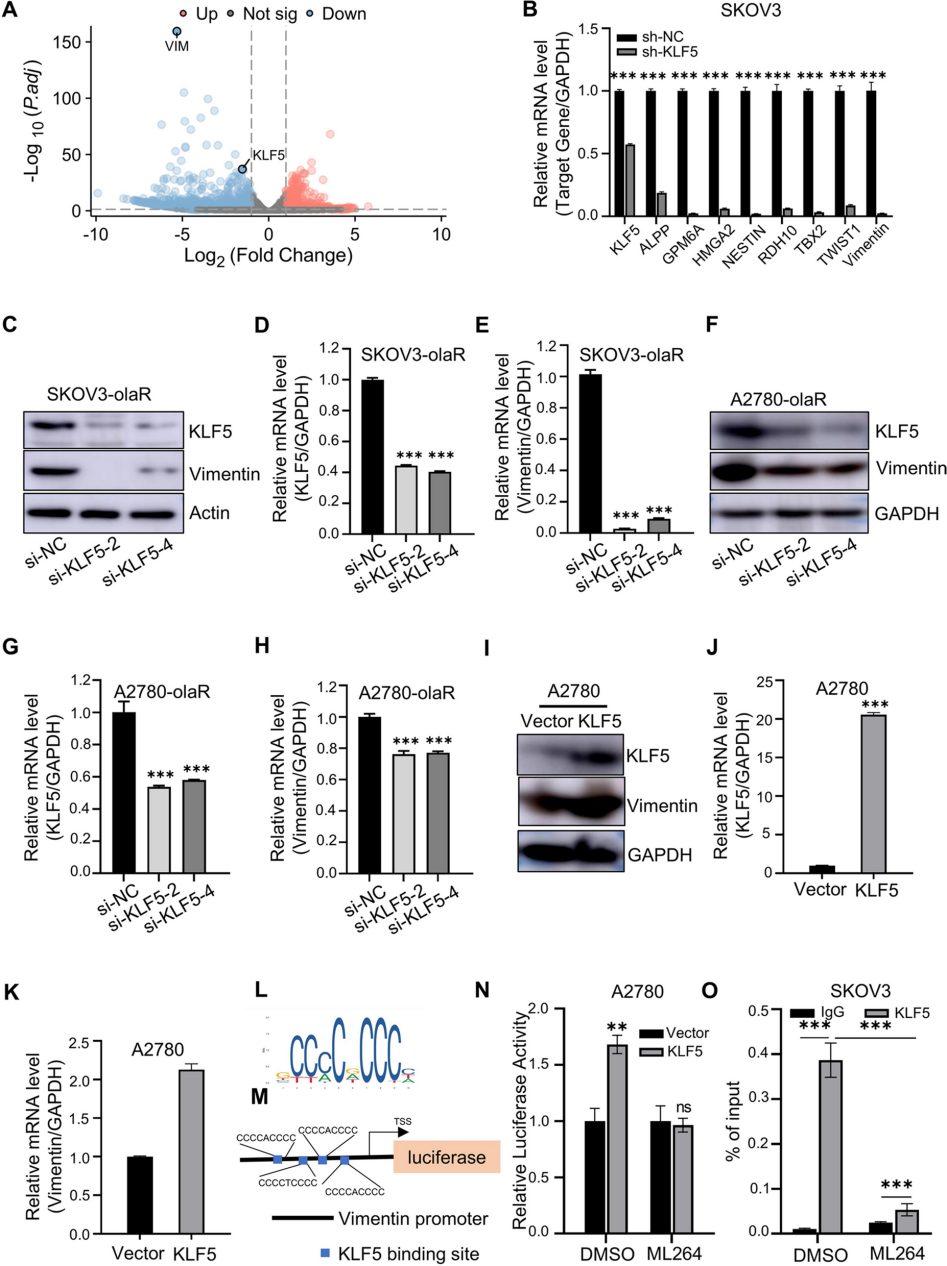
KLF5 regulates the expression of Vimentin. **A**. Volcano plot of differentially expressed genes in KLF5 knock down SKOV3 cells compared to corresponding control cells analyzed by RNA-Seq. **B** mRNA expression of genes in **A** was detected by qPCR in KLF5 knock down SKOV3 cells and corresponding control cells. **C** KLF5 and Vimentin protein expression detected by western blot in KLF5 knock down SKOV3-olaR cells and corresponding control cells. **D** KLF5 mRNA expression detected by qPCR in KLF5 knock down SKOV3-olaR cells and corresponding control cells. **E** Vimentin mRNA expression detected by qPCR in KLF5 knock down SKOV3-olaR cells and corresponding control cells. **F** KLF5 and Vimentin protein expression detected by western blot in KLF5 knock down A2780-olaR cells and corresponding control cells. **G** KLF5 mRNA expression detected by qPCR in KLF5 knock down A2780-olaR cells and corresponding control cells. **H** Vimentin mRNA expression detected by qPCR in KLF5 knock down A2780-olaR cells and corresponding control cells. **I** KLF5 and Vimentin protein expression detected by western blot in KLF5-overexpressing A2780 cells and corresponding control cells. **J** KLF5 mRNA expression detected by qPCR in KLF5-overexpressing A2780 cells and corresponding control cells. **K** Vimentin mRNA expression detected by qPCR in KLF5-overexpressing A2780 cells and corresponding control cells. **L** KLF5 binding motif predicted by JASPAR website. **M** Schematic diagram of Vimentin promoter (-1000-+100) construction onto the pGL4.26 plasmid with predicted KLF5 binding site shown. **N** Luciferase activity was measured in A2780 cells transfected with KLF5 overexpressing plasmid in combination with Vimentin promoter plasmid exposed to DMSO or ML264. **O** qPCR analysis of ChIP samples performed in SKOV3 cells exposed to DMSO or ML264. *P* value was obtained by Student’s t-test and ANOVA analysis. Results represent the mean ± SD of three independent experiments. *P < 0.05, **P < 0.01, ***P < 0.001

Stable transfected SKOV3 cell lines were generated using pLKO vectors to silence KLF5. High-throughput sequencing was conducted on SKOV3-sh-KLF5 and control cell lines to investigate downstream regulatory mechanisms associated with KLF5-induced ovarian cancer resistance to PARPi. RNA-seq analysis revealed the expression of 481 genes was up-regulated and 935 genes were down-regulated after KLF5 silencing (Fig. 6A). Among the genes that were down-regulated, we selected 9 genes KLF5, ALPP, GPM6A, HMGA2, NESTIN, RDH10, TBX2, TWIST1, and Vimentin for further verification by qPCR (Fig. 6B). Given the established association between Vimentin and tumor cell malignancy in numerous studies [26–28], Vimentin was chosen as the downstream gene of KLF5 in conjunction with RNA-seq data. Western blot and qPCR analysis further verified the decrease of Vimentin caused by KLF5 silencing (Fig. 6C-H). On the contrary, the overexpression of KLF5 resulted in a significant increase of Vimentin expression (Fig. 6I-K). To further demonstrate the regulatory role of KLF5 in Vimentin expression in ovarian cancer, JASPAR website was used to predict the binding motif of KLF5 and a Vimentin promoter was synthesized into pGL4.26 vector (Fig. 6L, M). Dual luciferase assays showed that overexpression of KLF5 promoted the activity of fluorescein in Vimentin promoter, which disappeared after the application of ML264 (Fig. 6N). These results demonstrated that transcription factor KLF5 interacted with the Vimentin Promoter to modulate its expression. Additionally, chromatin immunoprecipitation followed by qPCR further proved the binding of KLF5 on Vimentin, which was inhibited by ML264 (Fig. 6O). These findings provide evidence that Vimentin is a direct target gene of KLF5.

### KLF5 increases PARPi resistance and stemness in ovarian cancer through regulation of Vimentin

**Fig. 7:**
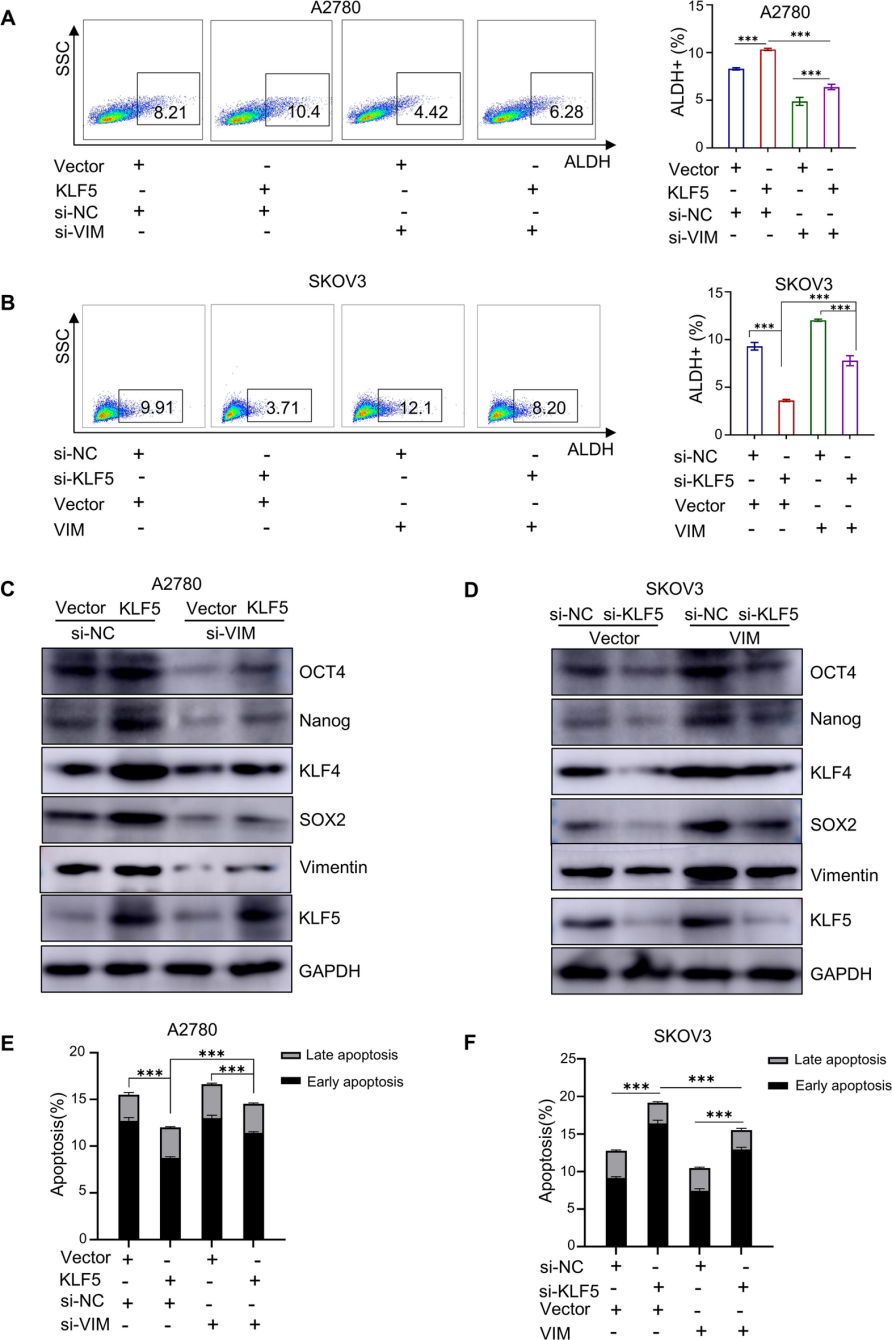
KLF5 increases stemness and PARPi resistance in ovarian cancer through regulation of Vimentin. **A**. Flow chart and statistical diagram of ALDH-positive cells showed loss of Vimentin could rescue the overexpression of KLF5 in stemness of A2780 cells. **B**. Flow chart and statistical diagram of ALDH-positive cells showed Vimentin could rescue the loss of KLF5 in stemness of SKOV3 cells. **C**. Western blot image of SOX2, KLF4, Nanog, OCT4, Vimentin and KLF5 protein expression with Vimentin-knocdown in KLF5-overexpressing A2780 cells and corresponding control cells. **D**. Western blot image of SOX2, KLF4, Nanog, OCT4, Vimentin and KLF5 protein expression with Vimentin-overexpression in KLF5-knockdown SKOV3 and corresponding control cells. **E**. Apotosis assay for investigating the potential of loss of Vimentin to rescue the overexpression of KLF5 in A2780 cells. **F**. Apotosis assay for investigating the potential of Vimentin to rescue the loss of KLF5 in SKOV3 cells.*P* value was obtained by ANOVA analysis. Results represent the mean ± SD of three independent experiments. *P < 0.05, **P < 0.01, ***P < 0.001

To investigate the role of Vimentin in the promotion of stemness and PARPi resistance in ovarian cancer by KLF5, we conducted Vimentin rescue experiments. Flow cytometry and western blot analysis suggested the enhanced stemness of ovarian cancer induced by KLF5 overexpression could be rescued following Vimentin knockdown (Fig. 7A, C). Conversely, the diminished stemness of ovarian cancer resulting from KLF5 knockdown could be rescued following Vimentin overexpression (Fig. 7B, D). Additionally, flow cytometry analysis revealed a notable decrease of apoptosis rates in A2780 cells exposed to olaparib due to overexpression of KLF5, which was rescued by knock down of Vimentin (Fig. 7E). On the contrary, Vimentin overexpression corrected the increase in olaparib-induced apoptosis caused by KLF5 knockdown in SKOV3 cells (Fig. 7F). Our findings suggested that KLF5 played a role in regulating PARPi resistance and stemness of ovarian cancer through the modulation of Vimentin expression.

## Discussion

The homologous recombination deficiency mechanism is involved in the repair of DNA double-strand breaks. Various PARPi have been developed based on the synthetic lethality effect resulting from the interaction between BRCA1/2 and PARP1 [10, 29]. In recent years, benefiting from clinical trials such as SOLO1, PAOLA-1, and PRIMA, multiple PARPi, including olaparib and niraparib, have been approved for clinical use [30–32]. The use of PARPi has undoubtedly benefited a large number of patients; however, similar to nearly all other targeted therapies, the development of resistance to PARPi treatment is foreseeable. This is indeed a complex issue that cannot be explained by a single mechanism. Research, not limited to ovarian cancer, has demonstrated these complex mechanisms, including but not limited to: upregulation of the drug efflux transporter ABCB1, which enhances drug efflux; [33] mutations in PARP1 that reduce the affinity for PARPi; [34] loss of poly(ADP-ribose) glycohydrolase (PARG) that restores poly(ADP-ribosyl)ation (PARylation) and mitigates synthetic lethality; [35] reversal or reactivation of BRCA1/2 genes; [36–40] BRCA-independent homologous recombination restoration; [41] and recovery of replication fork stability [42, 43]. Additionally, the team led by Wu was the first to discover that KLF5 can promote resistance to PARPi in ovarian cancer. This process occurs through the formation of a transcriptional complex by KLF5, which enhances the transcription of the RAD51 promoter, thereby strengthening the homologous recombination repair (HRR) pathway [25]. In this study, we focus on exploring how KLF5 modulates ovarian cancer stem cell properties to induce PARPi resistance.

KLF5 is widely recognized as a transcription factor that exerts oncogenic effects in various tumors and is associated with poor prognosis [4, 44, 45]. Numerous studies have also confirmed the close relationship between KLF5 and tumor cell stemness. In lung cancer, KLF5 mediates cell stemness promoted by α-Catulin; [46] in hepatocellular carcinoma, KLF5 overexpression enriches cancer stem cell-like populations, enhancing drug resistance; [47] in triple-negative breast cancer, metformin inhibits stemness in these cells by targeting KLF5 for degradation; [48] inflammatory cytokine TNF-α can partially promote basal-like breast cancer (BLBC) cell stemness through the activation of the KLF5-EphA2 axis; [49] and in colon cancer, miR-4711-5p suppresses tumor cell stemness by downregulating KLF5 expression through direct binding [50]. By comparing RNA-Seq data from PARPi-sensitive and resistant cell lines and conducting a series of experiments, we found that KLF5 is highly expressed in PARPi-resistant ovarian cancer cell lines. Furthermore, overexpression of KLF5 can promote the progression of ovarian cancer and PARPi resistance, which is consistent with Wu’s findings [25]. However, in this study, the results from spheroid formation assays and western blot analysis of stemness markers indicated a significant increase in stemness in PARPi-resistant ovarian cancer cells, representing an innovative discovery.

Cancer stem cells (CSCs) play a crucial role in treatment resistance, particularly in chemotherapy and radiotherapy, as they can accelerate tumor regeneration post-treatment [51, 52]. The proportion of CSCs within tumors can increase with tumor progression, and this increase is associated with treatment resistance [53, 54]. The mechanisms driving CSC resistance are complex, with epithelial-mesenchymal transition (EMT) being one of the primary factors. Additionally, hypoxia, oxidative regulation, autophagy, metabolic reprogramming, and epigenetic regulation also play important roles [55]. However, research related to PARPi resistance in this context has not yet been reported. Vimentin, a type III intermediate filament protein, is widely expressed in connective tissue cells and interfaces with various signaling pathways, regulating multiple cellular functions ranging from adhesion and differentiation to proliferation, migration, and invasion [28, 56–59]. It is upregulated during the EMT process in cancer metastasis and regulates angiogenesis, serving as a biomarker in various tumors [57, 60, 61]. In this study, Vimentin serves as an important bridge between KLF5 and tumor cell stemness. We demonstrated through database analysis and chromatin immunoprecipitation that KLF5 regulates Vimentin expression by binding to its promoter. A similar mechanism was observed in osteosarcoma, where KEAP1-259aa interacts with cytoplasmic Vimentin to regulate cell stemness, with Vimentin-induced multinucleation also affecting tumor cell stemness [62, 63]. 

While initial genetic manipulation has confirmed that inhibiting KLF5 can reduce the stemness of ovarian cancer cells and increase their sensitivity to PARPi, this study further explored the clinical application prospects of this mechanism using the KLF5 inhibitor ML264 for in vitro and in vivo validation. ML264 possesses excellent drug metabolism and pharmacokinetic characteristics, with potential high safety in terms of drug interactions [23]. In the animal experiments of this study, ML264 successfully reversed PARPi resistance to some extent and demonstrated good safety. However, it is regrettable that this study did not provide PDX samples for further validation. Interestingly, in vivo experiments, ML264 treatment did not lead to a significant reduction in KLF5 protein levels in tumor tissues, despite its clear inhibitory effects on tumor growth and PARPi resistance. This discrepancy may reflect differences in tumor type, tissue specificity, or in vivo pharmacodynamic response compared to previous reports [23]. In our study, the antitumor effect of ML264 may therefore be more closely related to the inhibition of KLF5 downstream functional pathways rather than direct suppression of its expression. Further studies are needed to clarify the context-dependent mechanisms of ML264 in different tumor models.

In summary, this study validates that KLF5 increases ovarian cancer cell stemness and induces PARPi resistance by binding to Vimentin and regulating its expression. It also preliminarily validates the potential of using ML264 in combination therapy for PARPi-resistant ovarian cancer, providing a new therapeutic strategy for the treatment of ovarian cancer.

## Electronic supplementary material

Below is the link to the electronic supplementary material.

Supplementary Material 1

Supplementary Material 2

Supplementary Material 3

Supplementary Material 4

Supplementary Material 5

Supplementary Material 6

Supplementary Material 7

Supplementary Material 8

## Data Availability

The datasets of this study are included within the article and the supplementary files, and part of the raw sequencing data generated in this study have been deposited in the NCBI GEO and under the accession number of GSE248659.

## References

[1] Zheng RS, Chen R, Han BF, Wang SM, Li L, Sun KX, et al. [Cancer incidence and mortality in China, 2022]. Zhonghua Zhong Liu Za Zhi. 2024;46(3):221–31. 38468501 10.3760/cma.j.cn112152-20240119-00035

[2] Siegel RL, Giaquinto AN, Jemal A. Cancer statistics, 2024. CA Cancer J Clin. 2024;74(1):12–49. 38230766 10.3322/caac.21820

[3] Webb PM, Jordan SJ. Global epidemiology of epithelial ovarian cancer. Nat Rev Clin Oncol. 2024;21(5):389–400. 38548868 10.1038/s41571-024-00881-3

[4] Wang C, Nie Z, Zhou Z, Zhang H, Liu R, Wu J, et al. The interplay between TEAD4 and KLF5 promotes breast cancer partially through inhibiting the transcription of p27Kip1. Oncotarget. 2015;6(19):17685–97. 25970772 10.18632/oncotarget.3779 PMC4627338

[5] Konstantinopoulos PA, Ceccaldi R, Shapiro GI, D’Andrea AD. Homologous recombination deficiency: exploiting the fundamental vulnerability of ovarian Cancer. Cancer Discov. 2015;5(11):1137–54. 26463832 10.1158/2159-8290.CD-15-0714 PMC4631624

[6] Ledermann J, Harter P, Gourley C, Friedlander M, Vergote I, Rustin G, et al. Olaparib maintenance therapy in platinum-sensitive relapsed ovarian cancer. N Engl J Med. 2012;366(15):1382–92. 22452356 10.1056/NEJMoa1105535

[7] Kim G, Ison G, McKee AE, Zhang H, Tang S, Gwise T, et al. FDA approval summary: Olaparib monotherapy in patients with deleterious germline BRCA-Mutated advanced ovarian Cancer treated with three or more lines of chemotherapy. Clin Cancer Res. 2015;21(19):4257–61. 26187614 10.1158/1078-0432.CCR-15-0887

[8] Tattersall A, Ryan N, Wiggans AJ, Rogozińska E, Morrison J. Poly(ADP-ribose) polymerase (PARP) inhibitors for the treatment of ovarian cancer. Cochrane Database Syst Rev. 2022;2(2):CD007929. 35170751 10.1002/14651858.CD007929.pub4 PMC8848772

[9] Li H, Liu Z-Y, Wu N, Chen Y-C, Cheng Q, Wang J. PARP inhibitor resistance: the underlying mechanisms and clinical implications. Mol Cancer. 2020;19(1):107. 32563252 10.1186/s12943-020-01227-0 PMC7305609

[10] Dias MP, Moser SC, Ganesan S, Jonkers J. Understanding and overcoming resistance to PARP inhibitors in cancer therapy. Nat Rev Clin Oncol. 2021;18(12):773–91. 34285417 10.1038/s41571-021-00532-x

[11] Saegusa M, Hashimura M, Yoshida T, Okayasu I. beta- Catenin mutations and aberrant nuclear expression during endometrial tumorigenesis. Br J Cancer. 2001;84(2):209–17. 11161379 10.1054/bjoc.2000.1581 PMC2363713

[12] Tsang YT, Deavers MT, Sun CC, Kwan S-Y, Kuo E, Malpica A, et al. KRAS (but not BRAF) mutations in ovarian serous borderline tumour are associated with recurrent low-grade serous carcinoma. J Pathol. 2013;231(4):449–56. 24549645 10.1002/path.4252 PMC4095747

[13] Wu R, Hendrix-Lucas N, Kuick R, Zhai Y, Schwartz DR, Akyol A, et al. Mouse model of human ovarian endometrioid adenocarcinoma based on somatic defects in the Wnt/beta-catenin and PI3K/Pten signaling pathways. Cancer Cell. 2007;11(4):321–33. 17418409 10.1016/j.ccr.2007.02.016

[14] Mor G, Yin G, Chefetz I, Yang Y, Alvero A. Ovarian cancer stem cells and inflammation. Cancer Biol Ther. 2011;11(8):708–13. 21317559 10.4161/cbt.11.8.14967 PMC3100563

[15] Hu L, McArthur C, Jaffe RB. Ovarian cancer stem-like side-population cells are tumourigenic and chemoresistant. Br J Cancer. 2010;102(8):1276–83. 20354527 10.1038/sj.bjc.6605626 PMC2856005

[16] Wilczyński JR, Wilczyński M, Paradowska E. Cancer stem cells in ovarian Cancer-A source of tumor success and a challenging target for novel therapies. Int J Mol Sci. 2022;23(5). 10.3390/ijms23052496 PMC8910575 35269636

[17] Steg AD, Bevis KS, Katre AA, Ziebarth A, Dobbin ZC, Alvarez RD, et al. Stem cell pathways contribute to clinical chemoresistance in ovarian cancer. Clin Cancer Res. 2012;18(3):869–81. 22142828 10.1158/1078-0432.CCR-11-2188 PMC3271164

[18] Dean M, Fojo T, Bates S. Tumour stem cells and drug resistance. Nat Rev Cancer. 2005;5(4):275–84. 15803154 10.1038/nrc1590

[19] Farrugia MK, Vanderbilt DB, Salkeni MA, Ruppert JM. Kruppel-like pluripotency factors as modulators of Cancer cell therapeutic responses. Cancer Res. 2016;76(7):1677–82. 26964625 10.1158/0008-5472.CAN-15-1806 PMC4873413

[20] Zeng L, Zhu Y, Moreno CS, Wan Y. New insights into KLFs and SOXs in cancer pathogenesis, stemness, and therapy. Semin Cancer Biol. 2023;90:29–44. 36806560 10.1016/j.semcancer.2023.02.003 PMC10023514

[21] Pan G, Thomson JA. Nanog and transcriptional networks in embryonic stem cell pluripotency. Cell Res. 2007;17(1):42–9. 17211451 10.1038/sj.cr.7310125

[22] Luo Y, Chen C. The roles and regulation of the KLF5 transcription factor in cancers. Cancer Sci. 2021;112(6):2097–117. 33811715 10.1111/cas.14910 PMC8177779

[23] Ruiz de Sabando A, Wang C, He Y, García-Barros M, Kim J, Shroyer KR, et al. ML264, A novel Small-Molecule compound that potently inhibits growth of colorectal Cancer. Mol Cancer Ther. 2016;15(1):72–83. 26621868 10.1158/1535-7163.MCT-15-0600 PMC4707060

[24] Bialkowska A, Crisp M, Madoux F, Spicer T, Knapinska A, Mercer B, et al. ML264: an antitumor agent that potently and selectively inhibits Krüppel-like factor five (KLF5) expression: A probe for studying Colon cancer development and progression. probe reports from the NIH molecular libraries program. Bethesda (MD): National Center for Biotechnology Information (US); 2010. 23762940

[25] Wu Y, Chen S, Shao Y, Su Y, Li Q, Wu J, et al. KLF5 promotes tumor progression and Parp inhibitor resistance in ovarian Cancer. Adv Sci (Weinh). 2023;10(31):e2304638. 37702443 10.1002/advs.202304638 PMC10625120

[26] Liu Ye, Zhao S, Chen Y, Ma W, Lu S, He L, et al. Vimentin promotes glioma progression and maintains glioma cell resistance to oxidative phosphorylation Inhibition. Cell Oncol (Dordr). 2023;46(6):1791–806. 37646965 10.1007/s13402-023-00844-3 PMC12974705

[27] Wang W, Zhu L, Zhou J, Liu X, Xiao M, Chen N, et al. Targeting the KRT16-vimentin axis for metastasis in lung cancer. Pharmacol Res. 2023;193:106818. 37315823 10.1016/j.phrs.2023.106818

[28] Tabatabaee A, Nafari B, Farhang A, Hariri A, Khosravi A, Zarrabi A, et al. Targeting vimentin: a multifaceted approach to combatting cancer metastasis and drug resistance. Cancer Metastasis Rev. 2024;43(1):363–77. 38012357 10.1007/s10555-023-10154-7

[29] Branco C, Paredes J. [PARP inhibitors: from the mechanism of action to clinical practice]. Acta Med Port. 2022;35(2):135–43. 35225777 10.20344/amp.13870

[30] Moore K, Colombo N, Scambia G, Kim B-G, Oaknin A, Friedlander M, et al. Maintenance Olaparib in patients with newly diagnosed advanced ovarian Cancer. N Engl J Med. 2018;379(26):2495–505. 30345884 10.1056/NEJMoa1810858

[31] González-Martín A, Pothuri B, Vergote I, DePont Christensen R, Graybill W, Mirza MR, et al. Niraparib in patients with newly diagnosed advanced ovarian Cancer. N Engl J Med. 2019;381(25):2391–402. 31562799 10.1056/NEJMoa1910962

[32] Ray-Coquard I, Leary A, Pignata S, Cropet C, González-Martín A, Marth C, et al. Olaparib plus bevacizumab first-line maintenance in ovarian cancer: final overall survival results from the PAOLA-1/ENGOT-ov25 trial. Ann Oncol. 2023;34(8):681–92. 37211045 10.1016/j.annonc.2023.05.005

[33] Vaidyanathan A, Sawers L, Gannon A-L, Chakravarty P, Scott AL, Bray SE, et al. ABCB1 (MDR1) induction defines a common resistance mechanism in paclitaxel- and olaparib-resistant ovarian cancer cells. Br J Cancer. 2016;115(4):431–41. 27415012 10.1038/bjc.2016.203 PMC4985349

[34] Pettitt SJ, Krastev DB, Brandsma I, Dréan A, Song F, Aleksandrov R, et al. Genome-wide and high-density CRISPR-Cas9 screens identify point mutations in PARP1 causing PARP inhibitor resistance. Nat Commun. 2018;9(1):1849. 29748565 10.1038/s41467-018-03917-2 PMC5945626

[35] Gogola E, Duarte AA, de Ruiter JR, Wiegant WW, Schmid JA, de Bruijn R et al. Selective loss of PARG restores parylation and counteracts PARP Inhibitor-Mediated synthetic lethality. Cancer Cell. 2018;33(6). 10.1016/j.ccell.2018.05.008 29894693

[36] Lin KK, Harrell MI, Oza AM, Oaknin A, Ray-Coquard I, Tinker AV, et al. BRCA reversion mutations in Circulating tumor DNA predict primary and acquired resistance to the PARP inhibitor Rucaparib in High-Grade ovarian carcinoma. Cancer Discov. 2019;9(2):210–9. 30425037 10.1158/2159-8290.CD-18-0715

[37] Weigelt B, Comino-Méndez I, de Bruijn I, Tian L, Meisel JL, García-Murillas I, et al. Diverse BRCA1 and BRCA2 reversion mutations in Circulating Cell-Free DNA of Therapy-Resistant breast or ovarian Cancer. Clin Cancer Res. 2017;23(21):6708–20. 28765325 10.1158/1078-0432.CCR-17-0544 PMC5728372

[38] Domchek SM. Reversion mutations with clinical use of PARP inhibitors: many genes, many versions. Cancer Discov. 2017;7(9):937–9. 28864639 10.1158/2159-8290.CD-17-0734

[39] Barber LJ, Sandhu S, Chen L, Campbell J, Kozarewa I, Fenwick K, et al. Secondary mutations in BRCA2 associated with clinical resistance to a PARP inhibitor. J Pathol. 2013;229(3):422–9. 23165508 10.1002/path.4140

[40] Norquist B, Wurz KA, Pennil CC, Garcia R, Gross J, Sakai W, et al. Secondary somatic mutations restoring BRCA1/2 predict chemotherapy resistance in hereditary ovarian carcinomas. J Clin Oncol. 2011;29(22):3008–15. 21709188 10.1200/JCO.2010.34.2980 PMC3157963

[41] Bouwman P, Aly A, Escandell JM, Pieterse M, Bartkova J, van der Gulden H, et al. 53BP1 loss rescues BRCA1 deficiency and is associated with triple-negative and BRCA-mutated breast cancers. Nat Struct Mol Biol. 2010;17(6):688–95. 20453858 10.1038/nsmb.1831 PMC2912507

[42] Rondinelli B, Gogola E, Yücel H, Duarte AA, van de Ven M, van der Sluijs R, et al. EZH2 promotes degradation of stalled replication forks by recruiting MUS81 through histone H3 trimethylation. Nat Cell Biol. 2017;19(11):1371–8. 29035360 10.1038/ncb3626

[43] Dungrawala H, Bhat KP, Le Meur R, Chazin WJ, Ding X, Sharan SK et al. RADX promotes genome stability and modulates chemosensitivity by regulating RAD51 at replication forks. Mol Cell. 2017;67(3). 10.1016/j.molcel.2017.06.023 PMC5548441 28735897

[44] Tong D, Czerwenka K, Heinze G, Ryffel M, Schuster E, Witt A, et al. Expression of KLF5 is a prognostic factor for disease-free survival and overall survival in patients with breast cancer. Clin Cancer Res. 2006;12(8):2442–8. 16638850 10.1158/1078-0432.CCR-05-0964

[45] Takagi K, Miki Y, Onodera Y, Nakamura Y, Ishida T, Watanabe M, et al. Krüppel-like factor 5 in human breast carcinoma: a potent prognostic factor induced by androgens. Endocr Relat Cancer. 2012;19(6):741–50. 22936544 10.1530/ERC-12-0017

[46] Tung C-H, Huang M-F, Liang C-H, Wu Y-Y, Wu J-E, Hsu C-L, et al. α-Catulin promotes cancer stemness by antagonizing WWP1-mediated KLF5 degradation in lung cancer. Theranostics. 2022;12(3):1173–86. 35154481 10.7150/thno.63627 PMC8771551

[47] Maehara O, Sato F, Natsuizaka M, Asano A, Kubota Y, Itoh J, et al. A pivotal role of Krüppel-like factor 5 in regulation of cancer stem-like cells in hepatocellular carcinoma. Cancer Biol Ther. 2015;16(10):1453–61. 26176896 10.1080/15384047.2015.1070992 PMC4846134

[48] Shi P, Liu W, Tala, Wang H, Li F, Zhang H, et al. Metformin suppresses triple-negative breast cancer stem cells by targeting KLF5 for degradation. Cell Discov. 2017;3:17010. 28480051 10.1038/celldisc.2017.10 PMC5396048

[49] Zhao P, Sun J, Huang X, Zhang X, Liu X, Liu R, et al. Targeting the KLF5-EphA2 axis can restrain cancer stemness and overcome chemoresistance in basal-like breast cancer. Int J Biol Sci. 2023;19(6):1861–74. 37063424 10.7150/ijbs.82567 PMC10092769

[50] Morimoto Y, Mizushima T, Wu X, Okuzaki D, Yokoyama Y, Inoue A, et al. miR-4711-5p regulates cancer stemness and cell cycle progression via KLF5, MDM2 and TFDP1 in colon cancer cells. Br J Cancer. 2020;122(7):1037–49. 32066912 10.1038/s41416-020-0758-1 PMC7109136

[51] Chou M-Y, Hu F-W, Yu C-H, Yu C-C. Sox2 expression involvement in the oncogenicity and radiochemoresistance of oral cancer stem cells. Oral Oncol. 2015;51(1):31–9. 25456004 10.1016/j.oraloncology.2014.10.002

[52] Luo M, Shang L, Brooks MD, Jiagge E, Zhu Y, Buschhaus JM et al. Targeting breast Cancer stem cell state equilibrium through modulation of redox signaling. Cell Metab. 2018;28(1). 10.1016/j.cmet.2018.06.006 PMC6037414 29972798

[53] Baumann M, Krause M, Hill R. Exploring the role of cancer stem cells in radioresistance. Nat Rev Cancer. 2008;8(7):545–54. 18511937 10.1038/nrc2419

[54] Iyer AK, Singh A, Ganta S, Amiji MM. Role of integrated cancer nanomedicine in overcoming drug resistance. Adv Drug Deliv Rev. 2013;65(13–14):1784–802. 23880506 10.1016/j.addr.2013.07.012

[55] Najafi M, Mortezaee K, Majidpoor J. Cancer stem cell (CSC) resistance drivers. Life Sci. 2019;234:116781. 31430455 10.1016/j.lfs.2019.116781

[56] Qin S, Jiang J, Lu Y, Nice EC, Huang C, Zhang J, et al. Emerging role of tumor cell plasticity in modifying therapeutic response. Signal Transduct Target Ther. 2020;5(1):228. 33028808 10.1038/s41392-020-00313-5 PMC7541492

[57] Chen Z, Fang Z, Ma J. Regulatory mechanisms and clinical significance of vimentin in breast cancer. Biomed Pharmacother. 2021;133:111068. 33378968 10.1016/j.biopha.2020.111068

[58] Usman S, Waseem NH, Nguyen TKN, Mohsin S, Jamal A, Teh M-T, et al. Vimentin is at the heart of epithelial mesenchymal transition (EMT) mediated metastasis. Cancers (Basel). 2021;13:19. 10.3390/cancers13194985 PMC8507690 34638469

[59] Berr AL, Wiese K, Dos Santos G, Koch CM, Anekalla KR, Kidd M, et al. Vimentin is required for tumor progression and metastasis in a mouse model of non-small cell lung cancer. Oncogene. 2023;42(25):2074–87. 37161053 10.1038/s41388-023-02703-9 PMC10275760

[60] Wu S, Du Y, Beckford J, Alachkar H. Upregulation of the EMT marker vimentin is associated with poor clinical outcome in acute myeloid leukemia. J Transl Med. 2018;16(1):170. 29925392 10.1186/s12967-018-1539-y PMC6009962

[61] Dave JM, Bayless KJ. Vimentin as an integral regulator of cell adhesion and endothelial sprouting. Microcirculation. 2014;21(4):333–44. 24387004 10.1111/micc.12111

[62] Kuburich NA, den Hollander P, Castaneda M, Pietilä M, Tang X, Batra H, et al. Stabilizing vimentin phosphorylation inhibits stem-like cell properties and metastasis of hybrid epithelial/mesenchymal carcinomas. Cell Rep. 2023;42(12):113470. 37979166 10.1016/j.celrep.2023.113470 PMC11062250

[63] Zhang Y, Liu Z, Zhong Z, Ji Y, Guo H, Wang W, et al. A tumor suppressor protein encoded by circKEAP1 inhibits osteosarcoma cell stemness and metastasis by promoting vimentin proteasome degradation and activating anti-tumor immunity. J Exp Clin Cancer Res. 2024;43(1):52. 38383479 10.1186/s13046-024-02971-7 PMC10880370

