# Supplementary figures and images for: Role of KLF5 in enhancing ovarian cancer stemness and PARPi resistance: mechanisms and therapeutic targeting
Source: J Transl Med. 2025 Apr 30;23:492. doi: 10.1186/s12967-025-06502-6 (PMC12042437; doi:10.1186/s12967-025-06502-6)

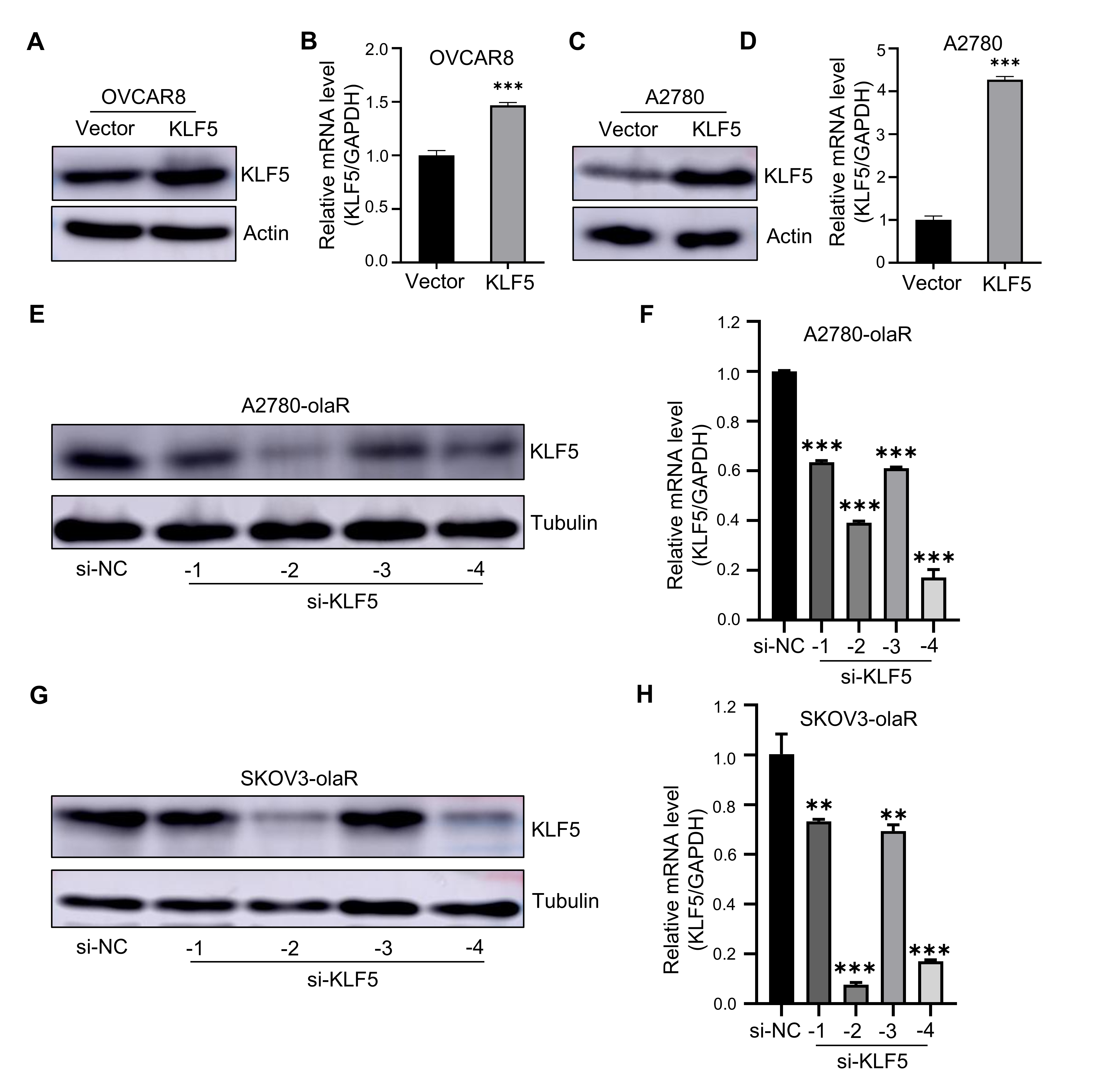

Supplement: Supplementary file 1 — Supplementary Material 1 [file 12967_2025_6502_MOESM1_ESM.tif]

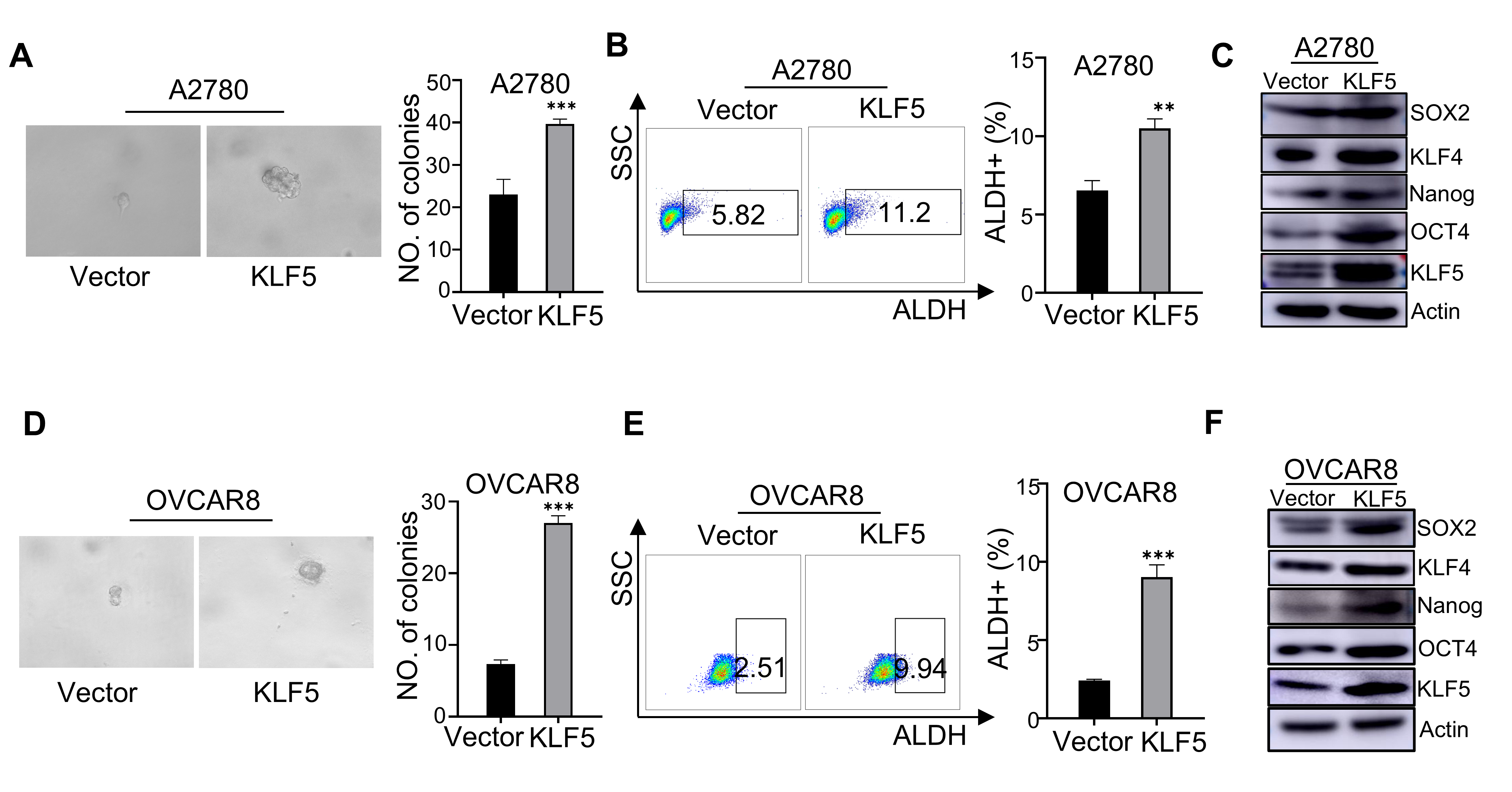

Supplement: Supplementary file 2 — Supplementary Material 2 [file 12967_2025_6502_MOESM2_ESM.tif]

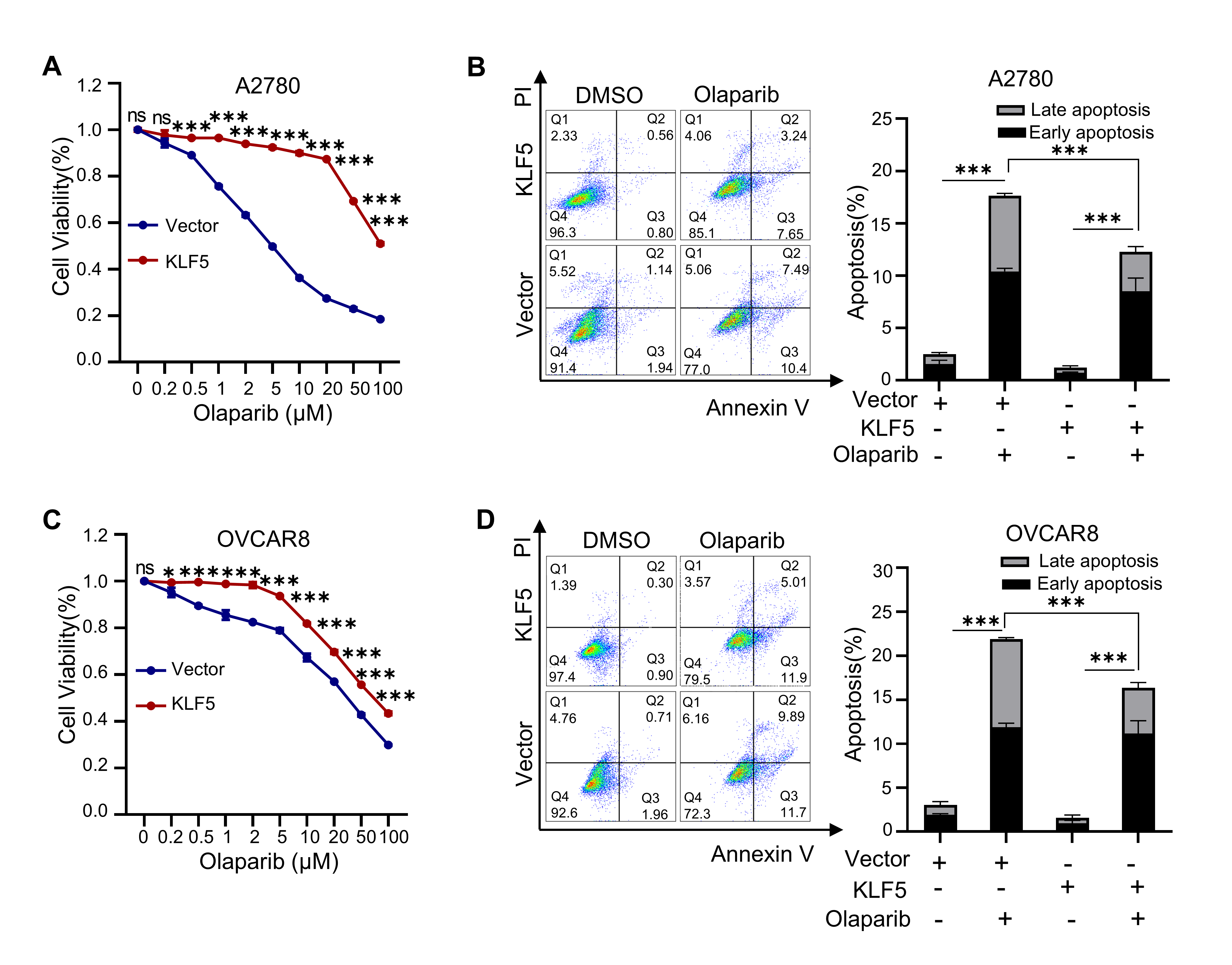

Supplement: Supplementary file 3 — Supplementary Material 3 [file 12967_2025_6502_MOESM3_ESM.tif]

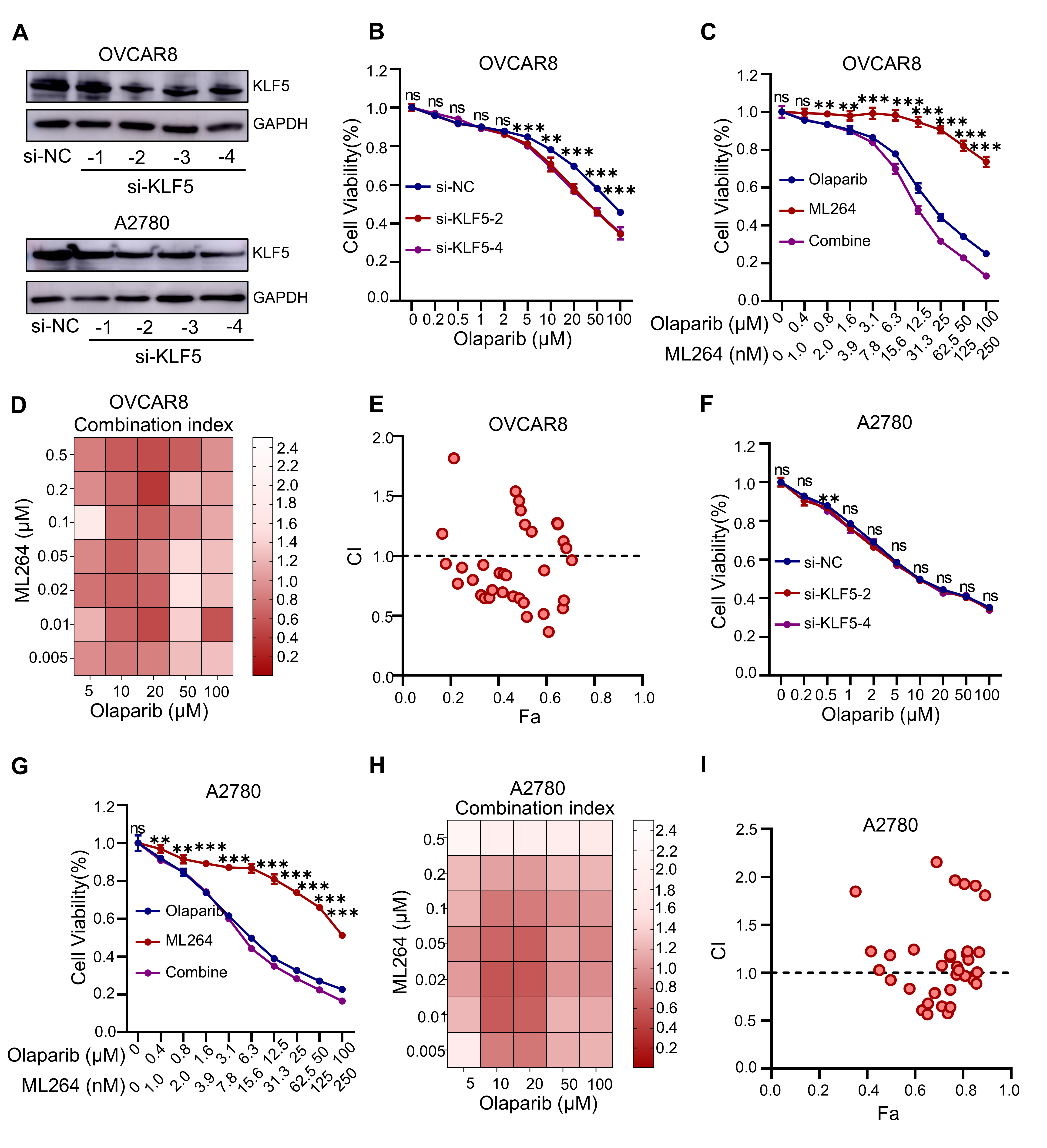

Supplement: Supplementary file 4 — Supplementary Material 4 [file 12967_2025_6502_MOESM4_ESM.tif]

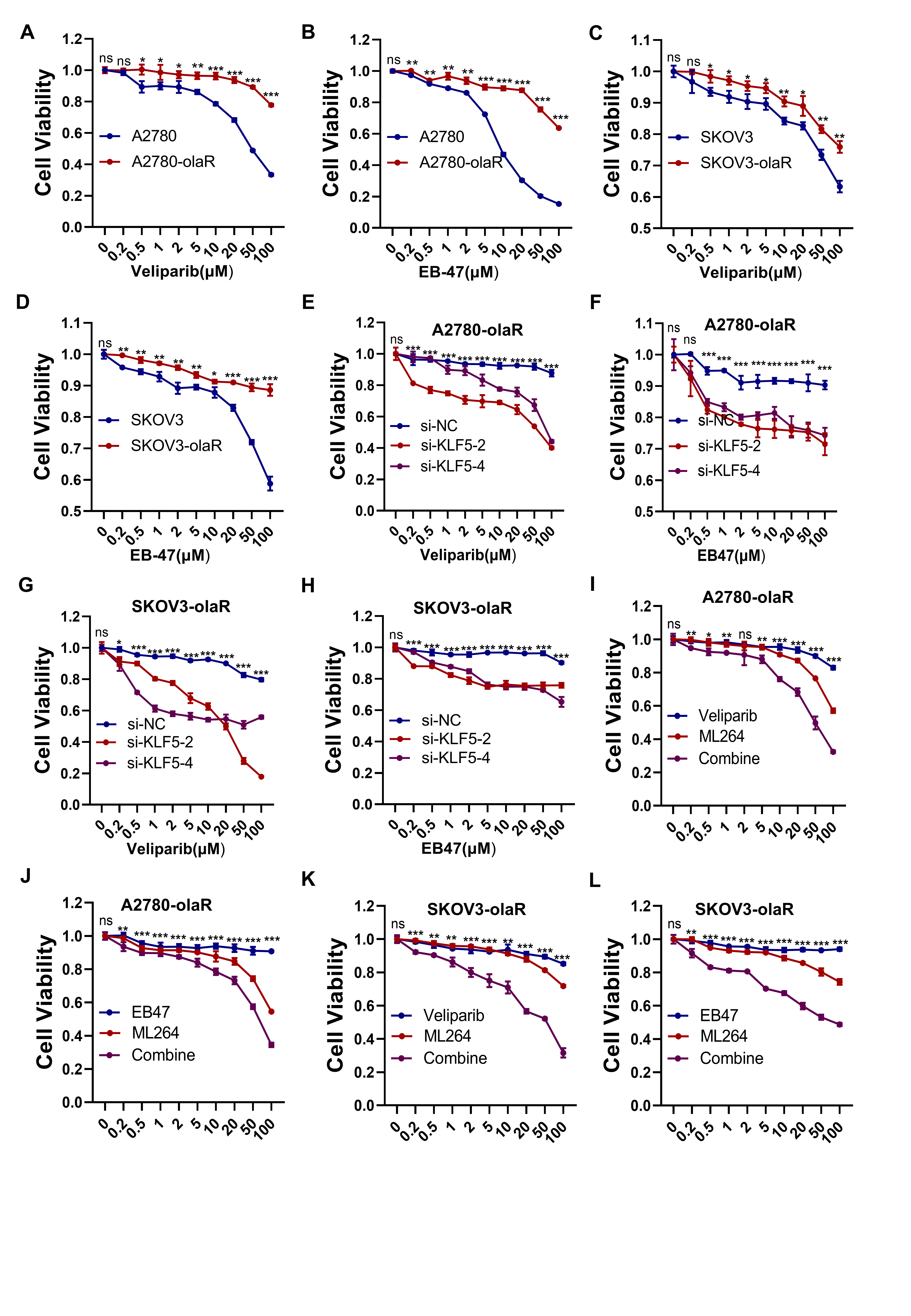

Supplement: Supplementary file 5 — Supplementary Material 5 [file 12967_2025_6502_MOESM5_ESM.tif]
