# Supplementary material for: Role of KLF5 in enhancing ovarian cancer stemness and PARPi resistance: mechanisms and therapeutic targeting
Source: J Transl Med. 2025 Apr 30;23:492. doi: 10.1186/s12967-025-06502-6 (PMC12042437; doi:10.1186/s12967-025-06502-6)
Supplement: Supplementary file 7 — Supplementary Material 7 [file 12967_2025_6502_MOESM7_ESM.docx]

## Supplementary Tables

### Table S1 Information of antibodies used in the study

| **Antibody** | **Application** | **Company** | **Catalog** | **RRID** | **Dilution** |
| --- | --- | --- | --- | --- | --- |
| KLF5 | WB, IHC | ABclonal | A2989 | AB_2764799 | WB:1:1000；IHC: 1:50 |
| KLF5 | ChIP | Abcam | Ab277773 |  | 1:100 |
| KLF4 | WB，IHC | Abclonal | A13673 | AB_2861693 | WB:1:1000；IHC: 1:50 |
| Nanog | WB | Abclonal | A22625 |  | 1:1000 |
| PARP1 | WB | Abclonal | A19596 | AB_2862689 | 1:1000 |
| Tubulin | WB | ABclonal | A12289 | AB_2861647 | 1:1000 |
| β-Actin | WB | ABclonal | AC038 | AB_2863784 | 1:1000 |
| Bcl-2 | WB | ABclonal | A196993 | AB_2862738 | 1:1000 |
| BAX | WB | ABclonal | A0207 | AB_2757021 | 1:1000 |
| Vimentin | WB | ABclonal | A19607 | AB_2862696 | 1:1000 |
| Caspase-3 | WB | CST | 9661 |  | 1:1000 |
| Caspase-3 | WB | ABclonal | A19654 | AB_2862718 | 1:1000 |
| OCT4 | WB | ABclonal | A7920 | AB_2770305 | 1:1000 |
| SOX2 | WB | ABclonal | 13647 |  | 1:1000 |
| GAPDH | WB | ABclonal | A19056 | AB_2862549 | 1:1000 |

### Table S2 Primer sequence of qPCR and ChIP -qPCR

| hKLF5-F | CAGGCCTTAACACACACACC |
| --- | --- |
| hKLF5-R | AGCAGCATAGGATGGAGGTG |
| hGAPDH-F | AGGTCGGAGTCAACGGATTT |
| hGAPDH-R | TGACGGTGCCATGGAATTTG |
| hVIM-F | GAGTCCACTGAGTACCGGAG |
| hVIM-R | GAGTCCACTGAGTACCGGAG |
| hALPP-F | GTCAGTGGGAGTGGTAACCA |
| hALPP-R | ACATGTACTTTCGGCCTCCA |
| hGPM6A-F | TCTGGTCACTCGCTCTCCTC |
| hGPM6A-R | CACCCTTTTTGTGTCTGTCCC |
| hHMGA2-F | CAGCAGCAAGAACCAACCG |
| hHMGA2-R | TCTTCGGCAGACTCTTGTGA |
| hNESTIN-F | ACCTCAAGATGTCCCTCAGC |
| hNESTIN-R | ACAGGTGTCTCAAGGGTAGC |
| hRDH10-F | CATGCACACTTCTGGACCAC |
| hRDH10-R | TCCATCCTTTTCAGCAGCCT |
| hTBX2-F | CCAGTTCCACAAGCTAGGCA |
| hTBX2-R | CATGCGTTTGGGCATCTCAG |
| hTWIST1-F | GCTGAGCAAGATTCAGACCCT |
| hTWIST1-R | CATCTTGGAGTCCAGCTCGT |
| ChIP -qPCR-F | CTGAAGTAACGGGACCATGC |
| ChIP -qPCR-R | GAGCGAGAGTGGCAGAGG |
