# Supplementary material for: Role of KLF5 in enhancing ovarian cancer stemness and PARPi resistance: mechanisms and therapeutic targeting
Source: J Transl Med. 2025 Apr 30;23:492. doi: 10.1186/s12967-025-06502-6 (PMC12042437; doi:10.1186/s12967-025-06502-6)
Supplement: Supplementary file 8 — Supplementary Material 8 [file 12967_2025_6502_MOESM8_ESM.docx]

**
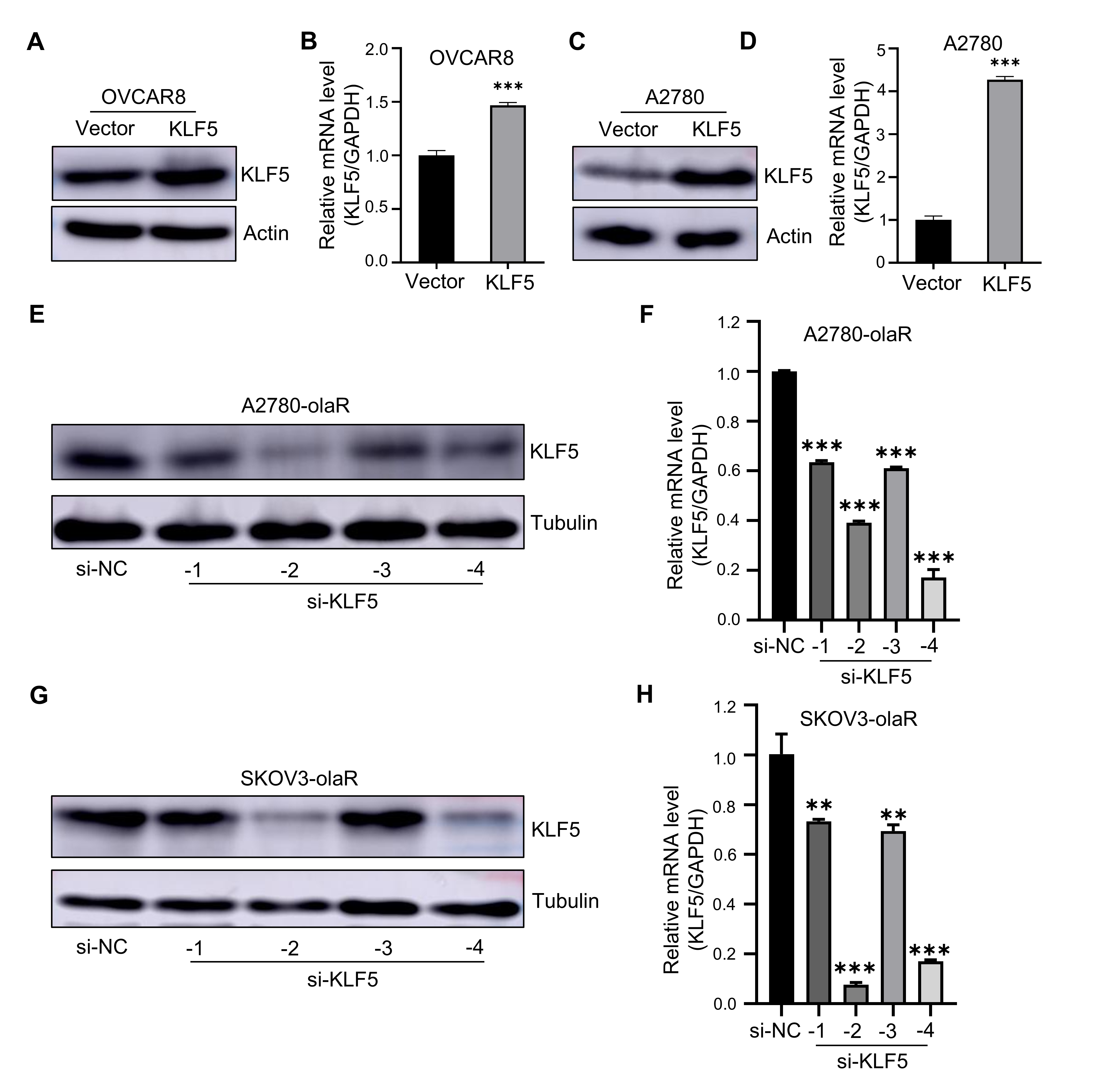
**

**SupFig 1. Establishment of KLF5 overexpression and knockdown cell lines.**

1. KLF5 protein expression detected by western blot in KLF5-overexpressing OVCAR8 cells and corresponding control cells.
2. KLF5 RNA expression detected by qPCR in KLF5-overexpressing OVCAR8 cells and corresponding control cells.
3. KLF5 protein expression detected by western blot in KLF5-overexpressing A2780 cells and corresponding control cells.
4. KLF5 RNA expression detected by qPCR in KLF5-overexpressing A2780 cells and corresponding control cells.
5. KLF5 protein expression detected by western blot in different KLF5 knock down A2780-olaR cells and corresponding control cells.
6. KLF5 RNA expression detected by qPCR in different KLF5 knock down A2780-olaR cells and corresponding control cells.
7. KLF5 protein expression detected by western blot in different KLF5 knock down SKOV3-olaR cells and corresponding control cells.
8. KLF5 RNA expression detected by qPCR in different KLF5 knock down SKOV3-olaR cells and corresponding control cells. *P* value was obtained by Student’s t-test and ANOVA analysis. Results represent the mean ± SD of three independent experiments. **P* < 0.05, ***P*< 0.01, ****P* < 0.001.


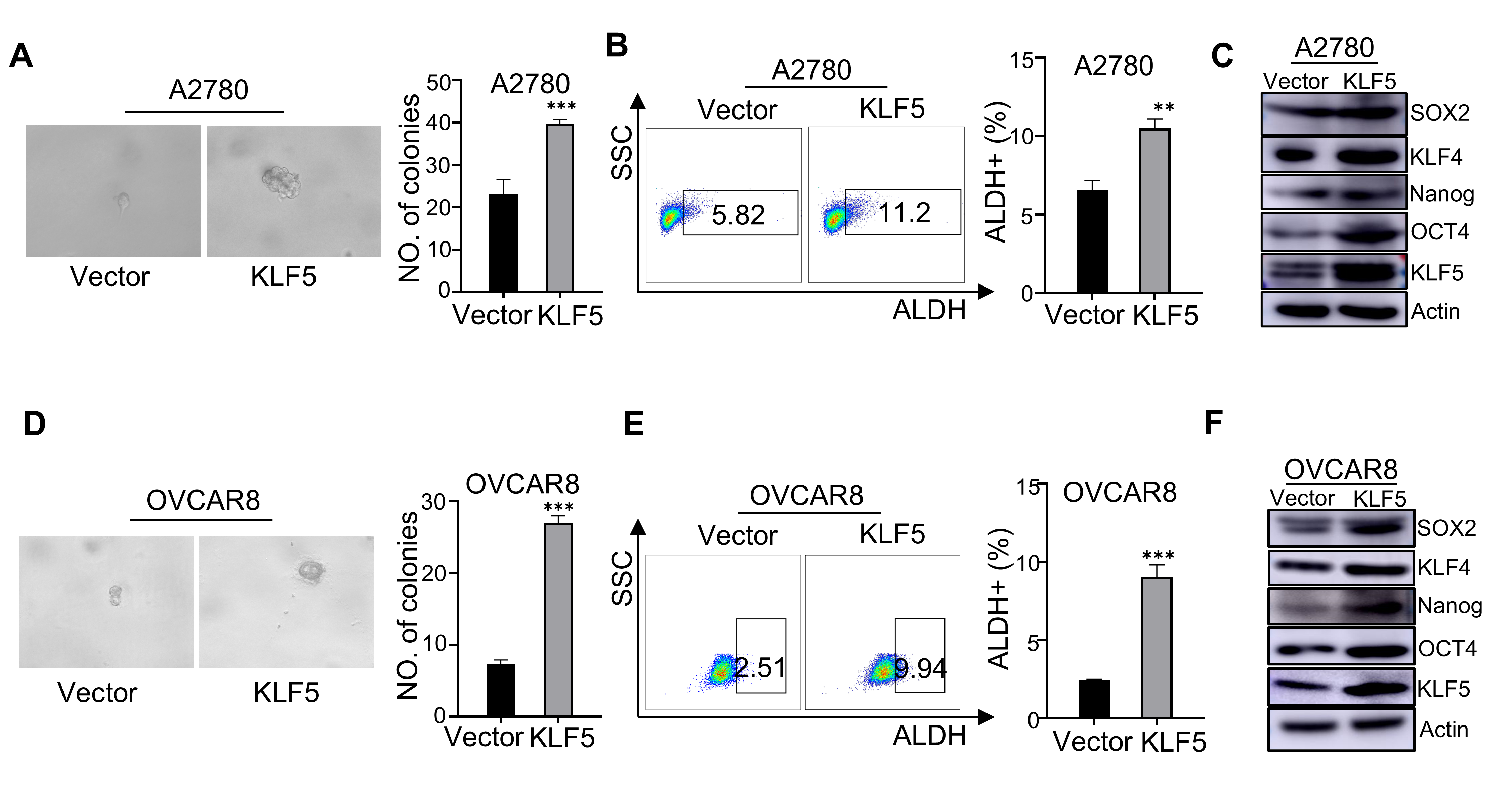


**SupFig 2. Overexpressing KLF5 increases the stemness of ovarian cancer.**

1. Representative pictures and statistical diagram of KLF5-overexpressing A2780 and corresponding control cell spheroidization.
2. Flow chart and statistical diagram of ALDH-positive cells in KLF5-overexpressing A2780 cells and corresponding control cells.
3. Western blot image of SOX2, KLF4, Nanog ,OCT4 and KLF5 protein expression in KLF5-overexpressing A2780 cells and corresponding control cells.
4. Representative pictures and statistical diagram of KLF5-overexpressing OVCAR8 cells and corresponding control cells spheroidization.
5. Flow chart and statistical diagram of ALDH-positive cells in KLF5-overexpressing OVCAR8 cells and corresponding control cells.
6. Western blot image of SOX2, KLF4, Nanog ,OCT4 and KLF5 protein expression in KLF5-overexpressing OVCAR8 cells and corresponding control cells.


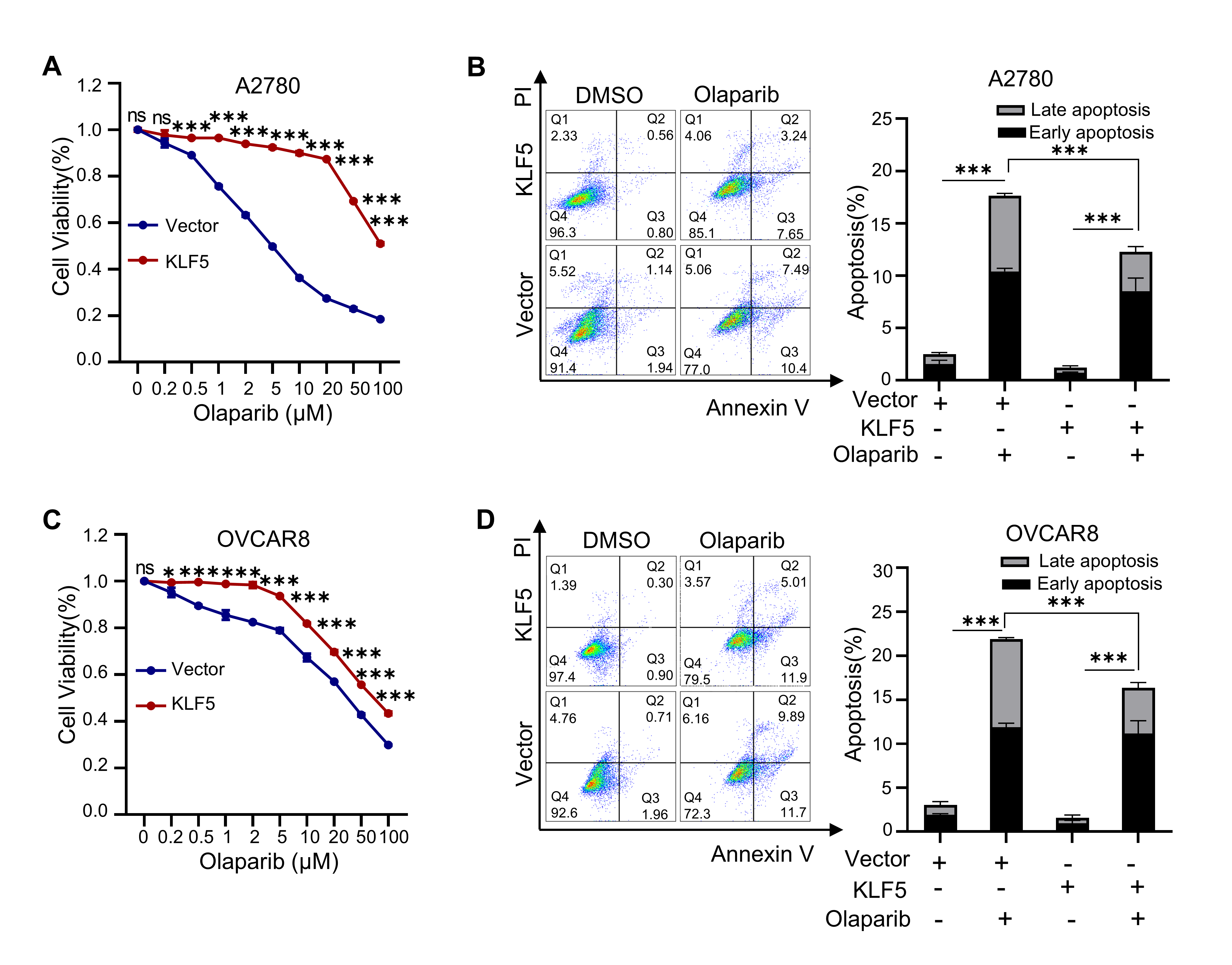


**SupFig 3.Overexpressing KLF5 reduces the sensitivity of PARPi-sensitive cells to PARPi in ovarian cancer.**

1. Cell viability curve of KLF5-overexpressing A2780 cells and corresponding control cells exposed to different concentrations of olaparib for 72h.
2. Flow chart and statistical diagram of apotosis cells in KLF5-overexpressing A2780 cells and corresponding control cells with DMSO or Olaparib.
3. Cell viability curve of KLF5-overexpressing OVCAR8 cells and corresponding control cells exposed to different concentrations of olaparib for 72h.
4. Flow chart and statistical diagram of apotosis cells in KLF5-overexpressing OVCAR8 cells and corresponding control cells with DMSO or Olaparib. *P* value was obtained by Student’s t-test and ANOVA analysis. Results represent the mean ± SD of three independent experiments. **P* < 0.05, ***P*< 0.01, ****P* < 0.001.


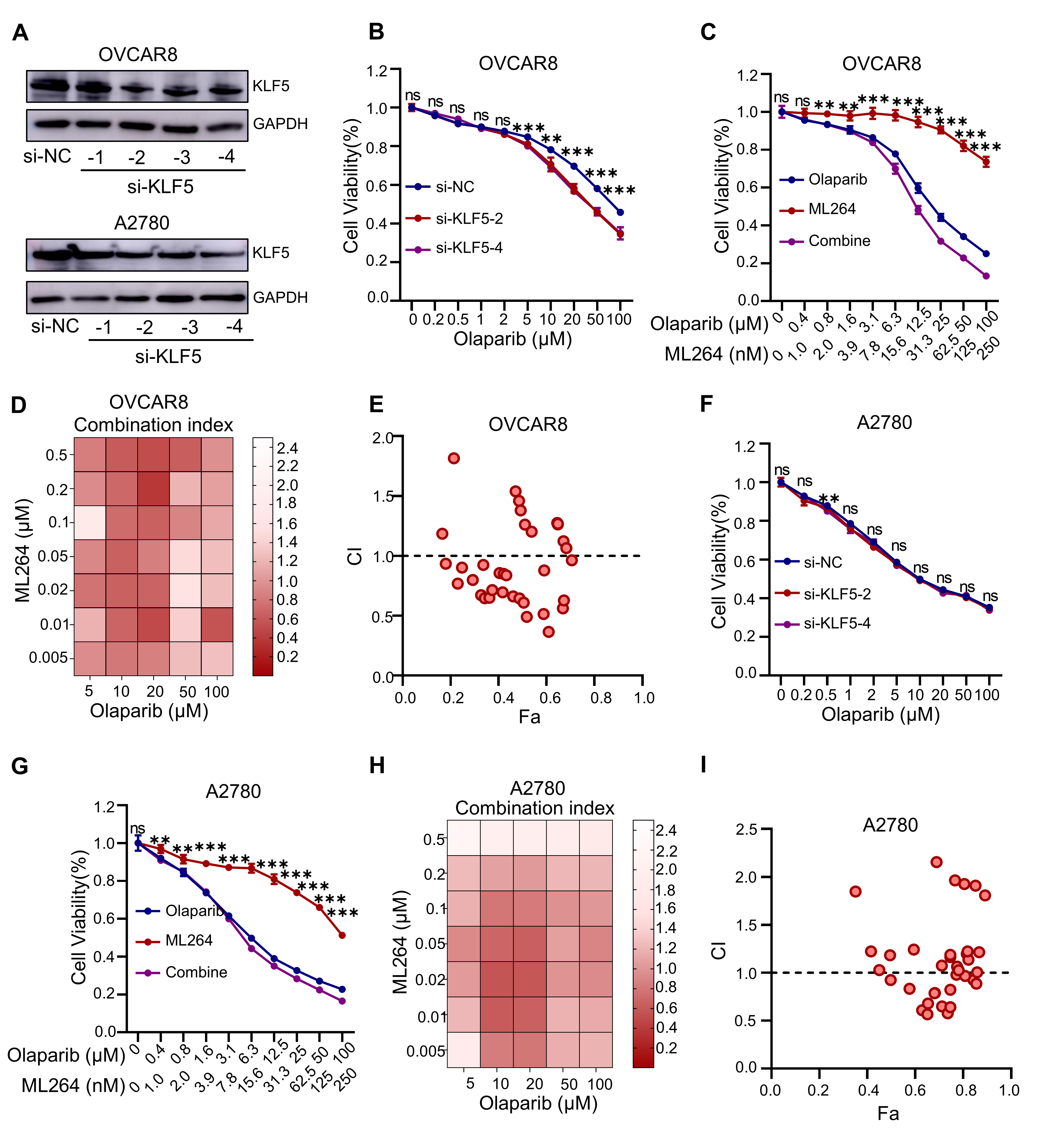


**SupFig4. Inhibiting KLF5 has minimal impact on the sensitivity of PARPi-sensitive cells to PARPi in ovarian cancer.**

1. KLF5 protein expression detected by western blot in different KLF5 knock down A2780 cells and OVCAR8 cells.

B. Cell viability curve of different KLF5 knock down A2780 cells exposed to different concentrations of olaparib for 72h.

C. Cell viability curve of A2780 cells exposed to different concentrations of olaparib, ML264 and the combination for 72h.

D. Heatmap of combination index (CI) values for combination treatment between olaparib and ML264 in A2780 cells.

E. CI values for the entire fraction affected (Fa) of A2780 cells.

F. Cell viability curve of different KLF5 knock down OVCAR8 cells exposed to different concentrations of olaparib for 72h.

G. Cell viability curve of OVCAR8 cells exposed to different concentrations of olaparib, ML264 and the combination for 72h.

H. Heatmap of combination index (CI) values for combination treatment between olaparib and ML264 in OVCAR8 cells.

I. CI values for the entire fraction affected (Fa) of OVCAR8 cells.


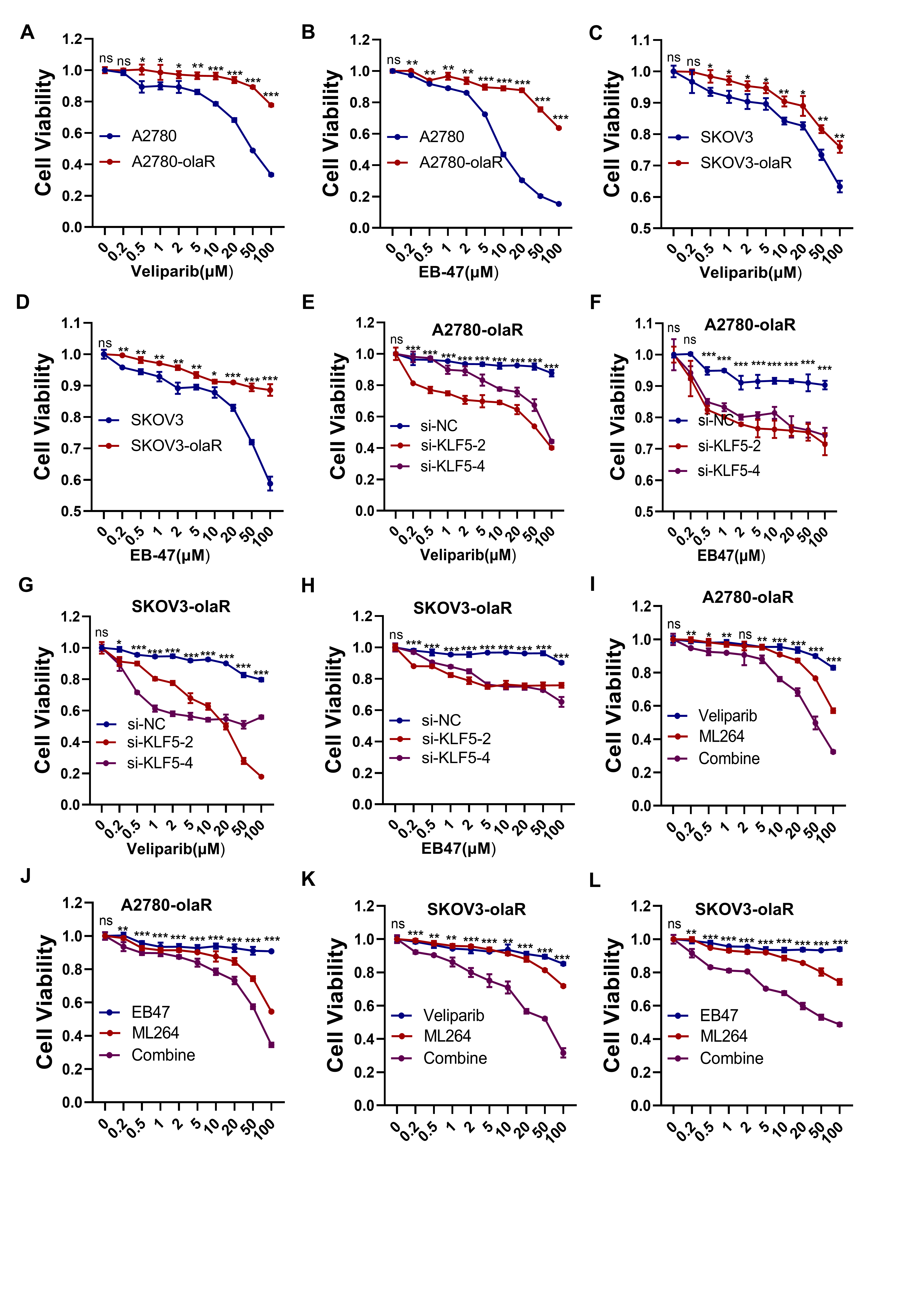


**SupFig5. Sensitivity curves of Veliparib and EB-47 across parental, PARPi-resistant, and KLF5-targeted ovarian cancer cell lines.**

A. Dose-response curve of Veliparib in A2780 and A2780-olaR (olaparib-resistant A2780) cell lines.

B. Dose-response curve of EB-47 in A2780 and A2780-olaR cell lines.

C. Dose-response curve of Veliparib in SKOV3 and SKOV3-olaR (olaparib-resistant SKOV3) cell lines.

D. Dose-response curve of EB-47 in SKOV3 and SKOV3-olaR cell lines.

E. Dose-response curve of Veliparib in A2780-olaR cells after KLF5 knockdown by dual siRNA sequences.

F. Dose-response curve of EB-47 in A2780-olaR cells after KLF5 knockdown by dual siRNA sequences.

G. Dose-response curve of Veliparib in SKOV3-olaR cells after KLF5 knockdown by dual siRNA sequences.

H. Dose-response curve of EB-47 in SKOV3-olaR cells after KLF5 knockdown by dual siRNA sequences.

I. Dose-response curve of Veliparib, ML264, and their combination in A2780-olaR cells.

J. Dose-response curve of EB-47, ML264, and their combination in A2780-olaR cells.

K. Dose-response curve of Veliparib, ML264, and their combination in SKOV3-olaR cells.

L. Dose-response curve of EB-47, ML264, and their combination in SKOV3-olaR cells.

**P* < 0.05, ***P*< 0.01, ****P* < 0.001.


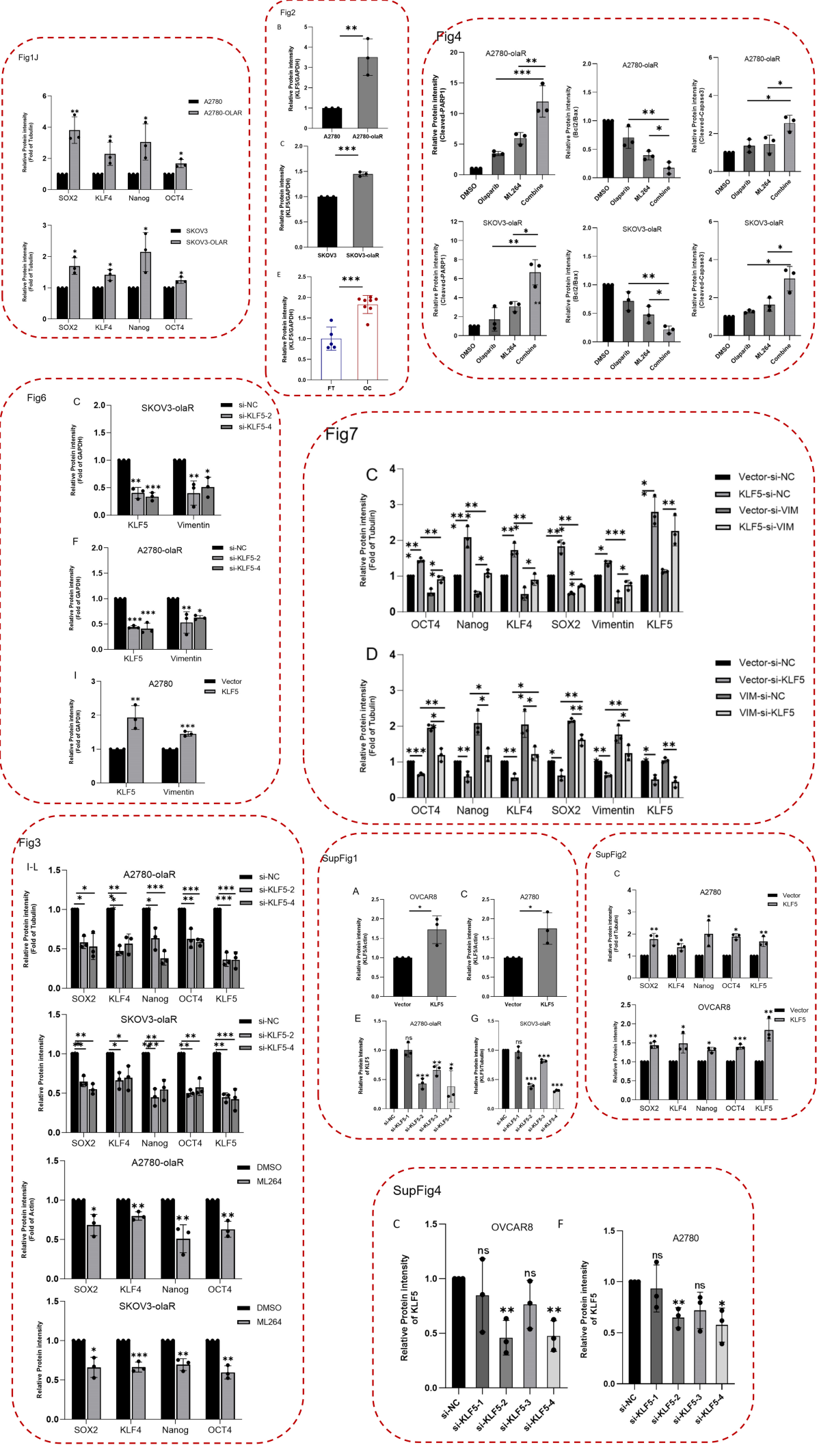


**SupFig6. Quantification of protein expression from Figures.**

This figure presents the statistical analysis of protein expression levels based on the densitometric quantification of Western blot results shown in Figures. Corresponding panel identifiers are indicated next to each bar graph to match the original figure panels. All data were derived from at least three independent experiments and are displayed as bar graphs for visual clarity.

**P* < 0.05, ***P*< 0.01, ****P* < 0.001.
